# Discovery and design of molecular glue enhancers of CDK12–DDB1 interactions for targeted degradation of cyclin K†

**DOI:** 10.1039/d4cb00190g

**Published:** 2024-10-11

**Authors:** Pompom Ghosh, Maximilian Schmitz, Thiyagamurthy Pandurangan, Solomon Tadesse Zeleke, Sean Chin Chan, John Mosior, Luxin Sun, Vinayak Palve, Dylan Grassie, Kanchan Anand, Sylvia Frydman, William R. Roush, Ernst Schönbrunn, Matthias Geyer, Derek Duckett, Andrii Monastyrskyi

**Affiliations:** a Department of Drug Discovery, Moffitt Cancer Center Tampa Florida 33612 USA andrii.monastyrskyi@moffitt.org; b Institute of Structural Biology, University of Bonn Venusberg-Campus 1 53127 Bonn Germany; c Department of Chemistry, The Scripps Research Institute Jupiter Florida 33458 USA

## Abstract

The CDK12 inhibitor SR-4835 promotes the proteasomal degradation of cyclin K, contingent on the presence of CDK12 and the CUL4–RBX1–DDB1 E3 ligase complex. The inhibitor displays molecular glue activity, which correlates with its enhanced ability to inhibit cell growth. This effect is achieved by facilitating the formation of a ternary complex that requires the small molecule SR-4835, CDK12, and the adaptor protein DDB1, leading to the subsequent ubiquitination and degradation of cyclin K. We have successfully solved the structure of the ternary complex, enabling the *de novo* design of molecular glues that transform four different CDK12 scaffold inhibitors, including the clinical pan-CDK inhibitor dinaciclib, into cyclin K degraders. These results not only deepen our understanding of CDK12's role in cell regulation but also underscore significant progress in designing molecular glues for targeted protein degradation in cancers associated with dysregulated cyclin K activity.

## Abstract 2

*De novo* design of molecular glues transforming CDK12 inhibitors, including dinaciclib, into cyclin K degraders.

## Graphical abstract

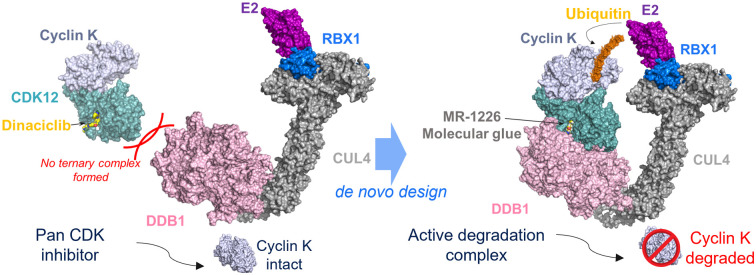

## Introduction

Cyclin-dependent kinases 12 and 13 (CDK12/13) and their cognate cyclin co-activator protein, cyclin K, regulate transcription elongation and termination, co-transcriptional splicing, as well as RNA turnover.^1–3^ CDK12/13 control these processes in part through phosphorylating the heptamer repeat at Ser2, Ser5 and Thr4 within the carboxy-terminal domain (CTD) of the largest subunit of RNA polymerase II (Pol II).^4^ The loss of CDK12/13 or cyclin K expression, impedes both global Pol II processivity and pre-mRNA processing.^5^ In particular, CDK12 has been shown to play essential roles in genome maintenance by regulating the expression of DNA damage repair (DDR) genes including *BRCA1* (breast and ovarian cancer type 1 susceptibility protein 1), *ATR* (ataxia telangiectasia and Rad3-related), *FANCI* (Fanconi anemia complementation groups – I) and *FANCD2* (Fanconi anemia complementation groups – D2).^6,7^ The molecular basis underpinning the selective effects of CDK12 silencing on DDR is consistent with defects in elongation due to premature cleavage and termination through increased processing at intronic polyadenylation (IPA) sites.^8,9^ IPA sites are enriched in longer genes, including DDR genes. Notably, the BRCAness phenotype, which is associated with hypersensitivity to poly (ADP–ribose) polymerase (PARP) inhibitors and DNA cross-linking agents extends the opportunity for similar synthetic-lethal strategies in CDK12 mutant tumors.^10–13^ Furthermore, tumors driven by oncogenes controlled by super-enhancers such as MYC and EWS/FLI1 are highly dependent on transcription and DDR gene expression for their rapid replication.^14^ Thus, inhibiting the function of CDK12 has been shown to be synthetically lethal with MYC overexpression (in neuroblastoma) and EWS/FLI1 expression (in Ewing sarcoma).^13,15–17^ Moreover, in human epidermal growth factor receptor (HER) 2-positive breast, gastric, colorectal, and papillary thyroid cancers a strong correlation between CDK12 level and high tumor grade exists.^18–21^ Together, these pre-clinical findings suggest that selective CDK12 and/or cyclin K degraders will not only serve as effective molecular tools to interrogate CDK12 biology, but might also be utilized alone or in combination with other treatments to combat cancer.

We recently reported an orally bioavailable CDK12/13 inhibitor, SR-4835, that selectively targets the CDK12/13 ATP binding site with high affinity.^22,23^ SR-4835 sensitizes triple-negative breast cancer cells to PARP inhibitors and DNA-damaging chemotherapeutics by reducing expression of the genes in the DNA damage response pathway and stimulates rapid tumor regression in multiple patient-derived xenograft mouse models. While other CDK12/CDK13 inhibitors, such as the covalent CDK12/13 inhibitor THZ531,^24^ have been reported, SR-4835 remains one of the few disclosed inhibitors with a set of pharmacokinetic properties suitable for *in vivo* studies. In recent findings, three independent research teams simultaneously identified small molecules that interact with the CDK12–cyclin K complex, acting as molecular glues to enhance the complex's stability with the CUL4–RBX1–DDB1 ubiquitin ligase complex.^25–27^ Remarkably, these agents facilitate cyclin K ubiquitination and its subsequent degradation by the proteasome without the need for a DCAF substrate receptor. Given these findings, we and others hypothesized that additional CDK12/13 inhibitors may also possess previously unrecognized capabilities as degraders,^28–30^ suggesting a broader application and potential for this class of inhibitors in therapeutic strategies.

In this study, we demonstrate that SR-4835 facilitates the proteasomal degradation of cyclin K *via* the establishment of CDK12 in complex with the CUL4–RBX1–DDB1 E3 ligase. We have successfully elucidated the crystal structure of the ternary DDB1ΔB·SR-4835·CDK12/CycK complex, which has enabled the *de novo* design of molecular glues capable of altering at least four distinct CDK12 scaffold inhibitors. In particular, we detail how introducing a surface-exposed benzimidazole moiety can strategically convert CDK12 inhibitors, including the widely used clinical pan-CDK inhibitor dinaciclib, into potent cyclin K degraders. These insights not only deepen our understanding of CDK12's regulatory roles but also reveal novel strategies for designing molecular glues aimed at targeted protein degradation—a complex and promising approach that could lead to more precise and effective strategies for targeting the ‘undruggable’ proteome.

## Results

### CDK12 inhibitor SR-4835 depletes cyclin K through proteasomal degradation

**Fig. 1:**
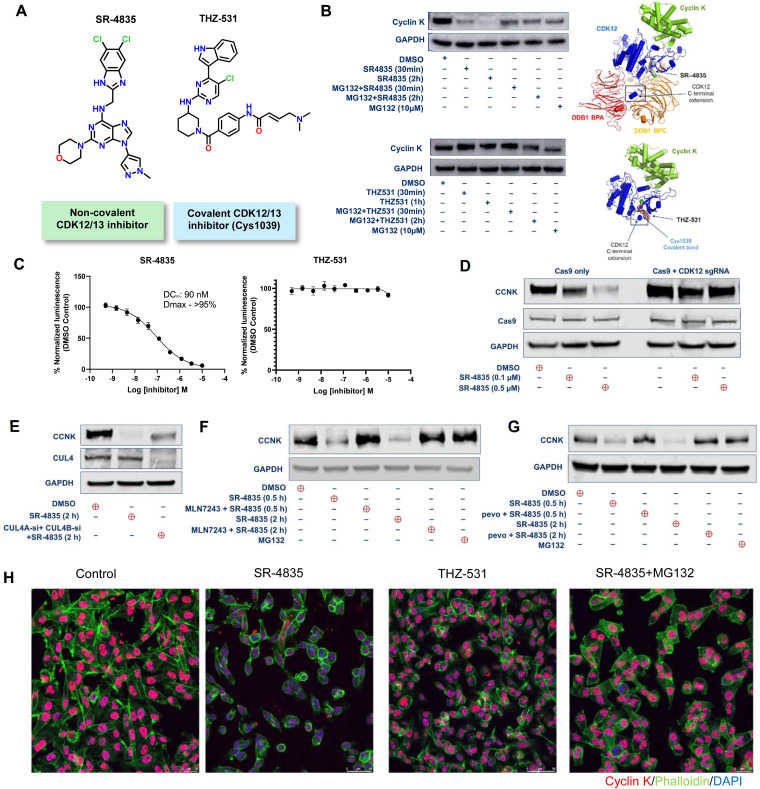
CDK12 inhibitor SR-4835 depletes cyclin K through proteasomal degradation and is dependent on the expression of CDK12 and the CUL4–RBX1–DDB1 E3 ligase complex. (A) Chemical structures of CDK12/13 inhibitors SR-4835 and THZ531. (B) Immunoblot depicting cyclin K levels in MDA-MB-231 cells treated with SR-4835 or THZ531 (500 nM) for the indicated time (left upper and lower panels) and docking poses of SR-4835 and THZ-531 in the active site of CDK12 (right upper and lower panels). (C) Degradation of HiBiT-tagged cyclin K using SR-4835 or THZ-531 (A549 cells). (D) Immunoblot analysis to assess if CDK12 is essential to provoke cyclin K degradation in the presence of SR-4835. (E) Transient knockdown of CUL4A/CUL4B gene expression led to reduced degradation of cyclin K upon treatment with SR-4835. (F) Rescue of cyclin K degradation by treatment of MBA-MD-231 cells with MLN7243; an inhibitor of the ubiquitin-activating enzyme. (G) Rescue of cyclin K degradation by treatment of MBA-MD-231 cells with pevonidistat (pevo), an inhibitor of NEDD8. (H) Immunofluorescence assay for visualizing loss of nuclear cyclin K upon treatment with SR-4835 (500 nM), THZ-531 (500 nM) and SR-4835 (500 nM) +MG132 (1 μM).

Previous studies have demonstrated that the CUL4–RBX1–DDB1 ubiquitin ligase complex facilitates cyclin K degradation in response to pan-CDK/CDK12 inhibitors, such as CR8, HQ461, and dCeMM2/3/4.^25–27^ To determine if SR-4835 promotes cyclin K degradation *via* similar mechanisms, MDA-MB-231 cells were treated with either SR-4835 or the covalent CDK12/13 inhibitor THZ531 (Fig. 1A) and assessed for cyclin K levels. THZ531 was used as a negative control as it is known to make a covalent bond with Cys1039 in a C-terminal extension of the kinase domain, thereby preventing this helix-loop-helix (HLH)-like fold from binding in a cleft between the DDB1 BPA and BPC domains (Fig. 1B, right panels). We observed that SR-4835, unlike THZ531, rapidly degrades cyclin K (Fig. 1B, left panels). As expected, pretreatment with the proteasomal inhibitor MG132 prevented cyclin K degradation even in the presence of SR-4835. An immunofluorescence-based assay developed for visualizing loss of nuclear cyclin K also confirmed the above observations (Fig. 1H and Fig. S1, ESI†).

Next, we utilized HiBiT technology (Promega) for quantitative analysis of cyclin K degrader activity. We conducted a dose–response analysis with SR-4835 and THZ531 in CRISPR-engineered cell lines (A549 cells with N-terminal HiBiT tag) to determine the concentration required for a 50% reduction in cyclin K levels (DC_50_). In comparison to the DMSO control, SR-4835 effectively degraded cyclin K (*D*_max_ > 95%) with a DC_50_ of approximately 90 nM after 2 hours, whereas THZ531 showed no reduction in cyclin K levels (Fig. 1C). Similar dose-dependent DC_50_ values were confirmed *via* immunoblotting at increasing concentrations of SR-4835 (0.01 to 3 μM) over 1 hour (Fig. S2, ESI†). Importantly, knockout of CDK12 using CRISPR/Cas9 protected cyclin K from SR-4835-induced degradation (Fig. 1D). We verified the depletion of CDK12 in HEK-293T cells (Fig. S3, ESI†).

Small molecule degraders typically function by chemically redirecting the cullin-RING family of E3 ubiquitin ligases (CRL). Key to this process, the paralogs CUL4A and CUL4B scaffold the assembly of the CRL complexes which are critical for regulating various chromatin-associated cellular functions. DDB1 functions as an adaptor protein for the CUL4A/B ligases and regulates biological processes such as nucleotide excision repair of DNA.^31^ Additionally, proteins such as RBX1, UBE2G1 (E2 ubiquitin-activating enzyme G1), and UBE2G2 interact with the cullin scaffold, catalyzing the formation of ubiquitin chains for proteasomal degradation. During ubiquitination, the cullin scaffold undergoes reversible neddylation facilitated by one E1 enzyme (NAE) and two E2 enzymes (UBE2M and UBE2F), along with several E3 enzymes. Notably, transient knockdown of CUL4A and CUL4B gene expression resulted in a significant reduction of cyclin K degradation following treatment with SR-4835, highlighting the mechanistic dependence of the compound efficacy on the presence and functionality of these ubiquitin ligase components (Fig. 1E). To further confirm if cyclin K depletion is mediated by the ubiquitin-proteasome system (UPS), MDA-MB-231 cells were pre-treated with inhibitors of the E1 ubiquitin-activating enzyme (MLN7243) and the NEDD8-activating enzyme (pevonedistat). While SR-4835 alone decreased cyclin K levels, the combined application of both inhibitors halted this effect, confirming that SR-4835 promotes cyclin K degradation through the UPS (Fig. 1F and G). Collectively, our results highlight that the molecular glue activity of SR-4835 is dependent upon a functional CUL4–RBX1–DDB1 ubiquitin ligase, and CDK12 is required for SR-4835-induced cyclin K degradation.

### The molecular glue activity of SR-4835 is correlated with its enhanced ability to inhibit cell growth

**Fig. 2:**
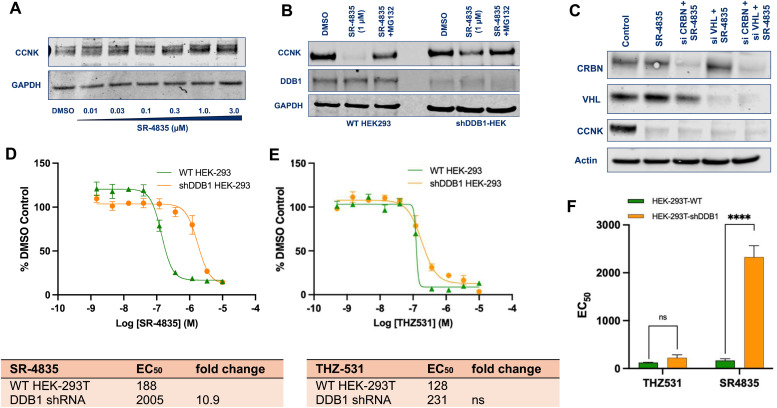
The molecular glue activity of SR-4835 is correlated with its cytotoxicity in HEK-293T cells. (A) Cyclin K levels remained unchanged in MDA-MB-231_DDB1-shRNA_ cells treated with SR-4835 (dose range 0.01 μM to 3 μM). (B) Cyclin K levels remained unchanged upon treatment with a single dose of SR-4835 (1 μM) in HEK-293T_DDB1-shRNA_ cells compared to WT-HEK-293T cells. (C) Immunoblots showing genetic ablation of VHL and CRBN do not rescue cyclin K degradation in contrast to loss of DDB1 which does inhibit cyclin K degradation. (D) and (E) CellTiter-Glo® viability assays comparing the EC_50_ values of SR-4835 and THZ531 in HEK-293T cells and its isogenic HEK-293T paired cell line with downregulated DDB1 expression (HEK-293T_DDB1-shRNA_). (F) Quantification of difference found in the EC_50s_ of THZ531 and SR-4835 in the HEK-293T and HEK-293T_DDB1-shRNA_ cells after 72 hours. Error bars indicate standard deviation (SD). Comparison between the indicated groups was performed by Student's un-paired two-tailed *t*-tests and statistical significance is expressed as ****, *p* < 0.0001 compared with HEK-293T-WT group.

To directly assess if DDB1 is necessary for the molecular glue function of SR-4835 we silenced the expression of DDB1 in both MDA-MB-231 and HEK293T cell lines. Notably, loss of DDB1 inhibited cyclin K degradation across a range of SR-4835 concentrations (Fig. 2A and B). Moreover, silencing DDB1 expression was sufficient to prevent cyclin K degradation and independent of CRBN or VHL (von Hippel-Lindau) expression (Fig. 2C). We next engineered HEK293T and MDA-MB-231 cell lines with reduced expression of DDB1. Assessment of compound activity in short-term cell growth assays of these paired isogenic cell lines highlights that DDB1 expression is necessary for the enhanced activity of SR-4835 but not for THZ531 (Fig. 2D–F; Fig. S4A–D, ESI†).

### Biochemical characterization of SR-4835's ability to induce interactions between Cdk12/CycK and DDB1

**Fig. 3:**
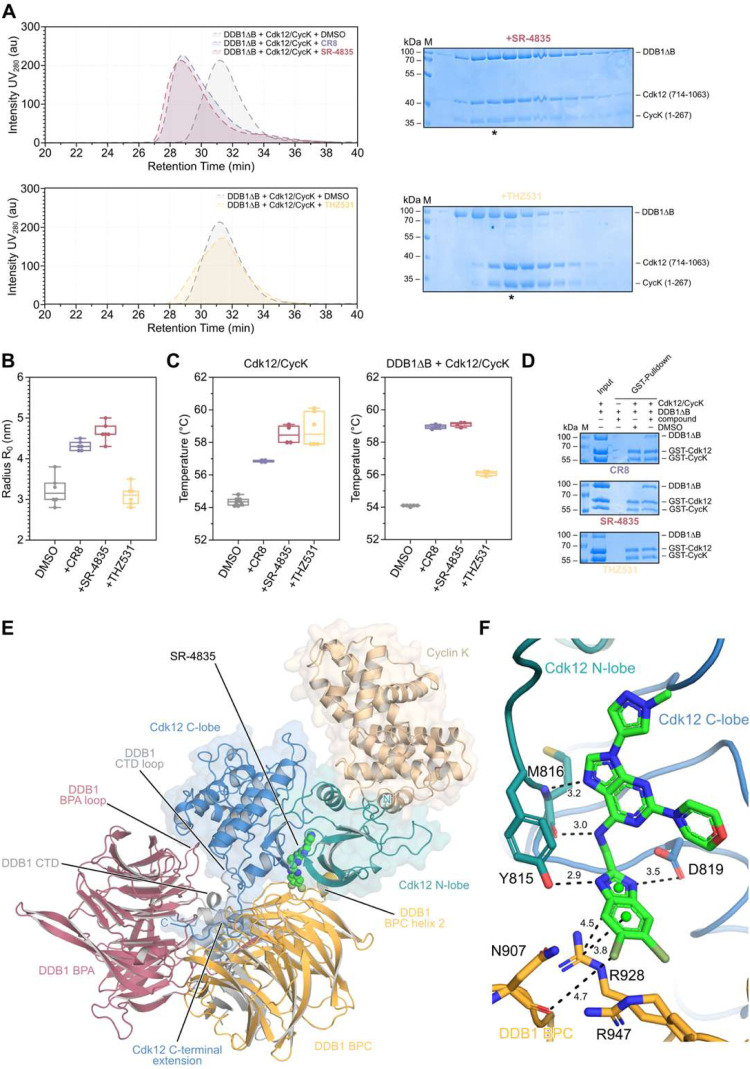
SR-4835 induces ternary complex formation between Cdk12/CycK and DDB1. (A) Analytical size exclusion chromatography of purified, recombinant DDB1ΔB and Cdk12/CycK proteins (20 μM) in presence of either 2% DMSO or 60 μM small molecular compounds CR8, SR-4835, or THZ531. Peak fractions were analyzed by SDS-PAGE, giving rise to a SR-4835-induced ternary complex between Cdk12/CycK and DDB1ΔB, compared to THZ531-treated negative control. Samples highlighted with asterisk were subsequently analyzed by DLS. (B) Dynamic light scattering analysis of peak middle fractions of previously conducted analytical size exclusion chromatography runs (in A). Hydrodynamic radii of CR8, and SR-4835-treated samples are significantly increased compared to negative controls, indicating ternary complex formation. Box-plots display the median of 6 data points per sample, each equivalent to the average of 20 DLS measurements. (C) Thermal stability analysis using the nanoDSF method of compound-treated Cdk12/CycK (left panel) and a mixture of compound-treated DDB1ΔB and Cdk12/CycK (right panel). All compounds including THZ531 stabilized Cdk12/CycK kinase. Degrader compounds elevated their stabilizing effects in presence of DDB1ΔB, indicating ternary complex formation, while THZ531 only stabilized Cdk12/CycK. (D) pulldown assays using GST-tagged variants of Cdk12 and CycK, identifying drug-induced recruitment of DDB1 in CR8- and SR-4835-treated samples. (E) Cartoon representation of the ternary complex structure of DDB1·SR-4835·Cdk12/CycK at a resolution of 3.9 Å (PDB: 9FMR). (F) Close-up of the SR-4835-linked interface between Cdk12 (C-lobe, blue; N-lobe, turquoise) and the DDB1ΔB BPC domain (bright orange), identifying key stacking interactions between the benzimidazole moiety of SR4835 with DDB1 residue R928. Key residues forming the interface are highlighted.

We investigated the ability of SR-4835 to induce the formation of protein–protein interactions between Cdk12/CycK and DDB1 by biochemical means, employing the previously characterized molecular glue degrader CR8^25^ as a positive control and the covalent inhibitor THZ531^24^ as a compound lacking gain-of-function abilities as a molecular glue degrader. Analytical size-exclusion chromatography (SEC) revealed degrader-triggered formation of higher molecular weight ternary complexes as evidenced by the reduced retention times of CR8 and SR-4835 treated samples compared to DMSO and THZ531 treated negative controls (Fig. 3A). The complex formation was further supported by SDS PAGE analysis, identifying fractions with a stoichiometric composition of both Cdk12/CycK and DDB1ΔB in samples treated with SR-4835 (Fig. 3A) and CR8 (Fig. S5, ESI†). Peak middle fractions of respective preceding SEC runs were further subjected to dynamic light scattering (DLS) analysis to determine the particle size distribution in solution. Here, degrader-treated samples displayed significantly increased hydrodynamic radii compared to equivalent negative control samples, indicative for the formation of drug-induced protein–protein complexes (Fig. 3B).

Thermal stability measurements identified SR-4835's potential to stabilize the heterodimeric Cdk12/CycK complex elevating the melting temperature by +4.1 °C (Fig. 3C, left panel; Fig. S5B, ESI†). The complex stability was even further increased in samples comprising both Cdk12/CycK and DDB1ΔB proteins (Δ*T*_m_ = +5.05 °C) (Fig. 3C, right panel; Fig. S5B, ESI†), indicating drug-dependent interactions between these proteins. The same trend was also observed for CR8 treated samples. In contrast, THZ531 treatment, albeit leading to significant elevations of Cdk12/CycK's melting temperature (Δ*T*_m_ = +4.15 °C) (Fig. 3C, left panel; Fig. S5B, ESI†), demonstrated diminished thermal stabilizing effects on protein mixtures containing both Cdk12/CycK and DDB1ΔB (Δ*T*_m_ = +2 °C) (Fig. 3C, right panel; Fig. S5B, ESI†), suggesting exclusive engagement of THZ531 with Cdk12/CycK. All tested compounds displayed no stabilizing effect on the DDB1ΔB moiety alone, but either had no influence on the melting temperature or even moderately destabilized the protein (Fig. S5C, ESI†).

The drug-dependent recruitment of DDB1ΔB to Cdk12/CycK was further identified in pulldown experiments, using GST-tagged variants of Cdk12 and CycK as the bait moiety (Fig. 3D). There, CR8 and SR-4835 treatment led to the enrichment of DDB1ΔB fractions compared to DMSO-containing bead binding control samples, whereas THZ531 was incapable of recruiting the DDB1ΔB protein to the immobilized Cdk12/CycK kinase complex.

Finally, the capacity of small molecules to induce a ternary complex between CDK12/cyclin K and DDB1 *in vitro* was assessed by microscale thermophoresis (MST). Labeled DDB1ΔBPB was combined with purified CDK12/cyclin K, and fluorescence intensity was monitored with increasing compound concentrations. SR-4835 successfully prompted a ternary complex with an average dissociation constant (*K*_d_) of 54.3 ± 5.4 nM, whereas THZ-531 demonstrated an inability to induce such a complex (Fig. S6, ESI†). Notably, dinaciclib, too, lacked the ability to induce a ternary complex, and SR-4835 did not show binding potential for DDB1 in the absence of CDK12/cyclin K. These studies confirm that tertiary structure formation is dependent on the presence of SR-4835, DDB1 and CDK12/cyclin K.

### Crystal structure of the DDB1ΔB·SR-4835·Cdk12/CycK complex

To understand how SR-4835 facilitates the interaction between Cdk12/CycK and DDB1ΔB, crystallization trials were conducted and the X-ray crystal structure of the SR-4835 induced tripartite protein complex determined. The structure at 3.9 Å resolution revealed a linear assembly of DDB1ΔB with Cdk12/CycK in which SR-4835 establishes distinct contacts between the kinase and the adaptor protein (Fig. 3E), similar to the assembly induced by CR8.^25,29^ SR-4835 partially occupies the kinase active site and interacts extensively with the kinase hinge region as previously observed in the SR-4835·Cdk12/CycK structure.^23^ The benzimidazole moiety, which is surface exposed in the structure containing only the CDK/cyclin complex, forms direct contacts to residues within the β-propeller domain C (BPC) of DDB1 in the ternary complex, thereby facilitating the interaction between DDB1ΔB and Cdk12. The N- and C-terminal lobes of Cdk12 establish further extensive protein–protein interactions with DDB1, sharing an interface area of approximately 4,300 Å^2^, counting both molecules. These additional compatible interfaces are mainly formed in between the N-lobe of Cdk12 and residues within the DDB1 helix 2 of BPC, and the Cdk12 C-lobe and residues within DDB1's BPA and C-terminal loops. Additionally, the Cdk12 C-terminal extension helix that is visible up to residue 1047 in the electron density map, is displaced from the active site cleft, contrasting previously solved Cdk12/CycK structures.^4,24,32^ In the ternary complex structure, the C-terminal extension helix is located in a cleft formed in between the BPA and BPC domains of DDB1, engaging with residues proximal to the cleft.

As previously described, SR-4835's central purine moiety is bound within a specific hydrogen bond network facilitated by the kinase hinge region.^23^ Additionally, the dichloro-benzimidazole moiety is positioned by flanking hydrogen bonds established by the side chains of Cdk12 residues Y815 and D819 (Fig. 3F). In the ternary complex structure, this dichloro-benzimidazole unit forms distinct contacts with residues within DDB1, comprising a buried surface area (BSA) of approximately 180 Å^2^. The DDB1–Cdk12 interface is bridged by π–cation stacking interactions between the aromatic ring system of SR-4835 and key residue R928 of DDB1, similarly as observed in the CR8 containing ternary complex structure.^25^ Furthermore, the benzimidazole moiety facilitates contacts to R947 (BSA 20.6 Å^2^) and N907 (BSA 27.4 Å^2^), potentially forming a halogen bond with the backbone carbonyl bond (Fig. 3F).

The overall assembly suggests that Cdk12 acts as a glue-triggered substrate receptor that positions CycK in a manner similar to endogenous substrates that are recruited to the E3 ligase system for ubiquitination and subsequent proteasome-specific degradation. Notably, the crystal structure resolved in our study was also recently determined by Kozicka and colleagues, who identified 28 different compound-induced complexes between DDB1 and CDK12/cyclin K. Our findings further reiterate and strengthen confidence in the structural bases of small molecule CDK12/cycK–DDB1 ternary complex formation driving the degradation of cycK.^29^

### SR-4784 and its photoactivatable analog, SR-4784-PAP, require DDB1 to facilitate proximity between DDB1, CDK12, and cyclin K

**Fig. 4:**
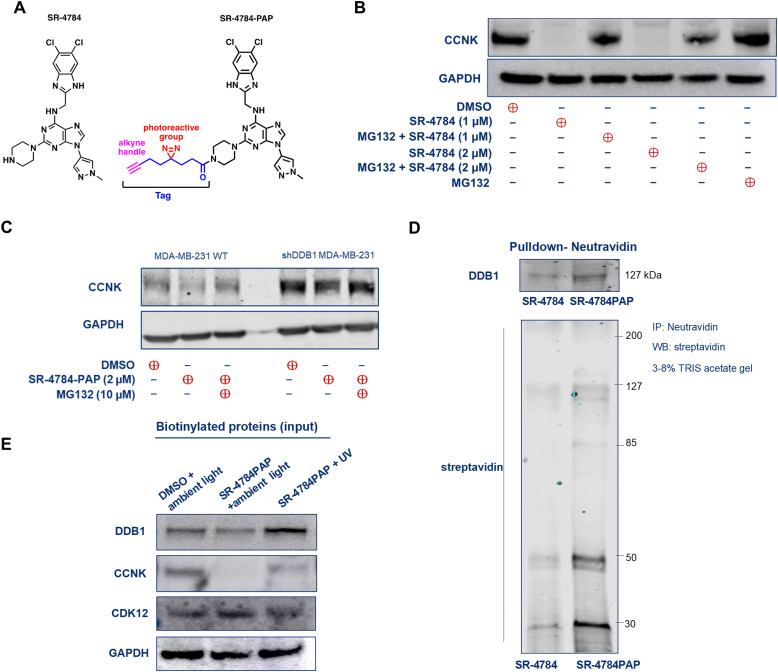
SR-4784 and SR-4784-PAP require DDB1 to degrade cyclin K and induce proximity between DDB1–CDK12–cyclin K. (A) Chemical structures of CDK12/13 inhibitors SR-4784 and SR-4784-PAP. (B) Immunoblot depicting cyclin K levels in MDA-MB-231 cells treated with SR-4784 alone (1–2 μM for 2 hours) and/or with MG132 (1 μM for 30 minutes). (C) Immunoblots showing cyclin K degradation in parent WT MDA-MB-231 and MDA-MB-231_DDB1-shRNA_ cell lines exposed to SR-4784-PAP alone (for 2 hours at 2 μM) or with MG132 (1 μM for 30 minutes). (D) Neutravidin agarose pull-down shows DDB1 enrichment by SR-4784PAP compared to SR-4784. Detection of biotinylated proteins by anti-streptavidin antibody and DDB1 is enriched in the SR-4784-PAP treated sample (for 2 hours at 2 μM). (E) Immunoblots of biotin-labelled proteins after click chemistry reaction showing cyclin K, CDK12, and DDB1 levels (for 2 hours at 2 μM).

To determine if SR-4835-induced cyclin K degradation is mediated through direct engagement with CRL4-E3 ligases, we conducted drug affinity pulldown experiments, as described previously.^27^ For in-cell drug-target engagement assays, SR-4784 was modified with a photoactive diazirine group and an alkyne handle, creating SR-4784-PAP (Fig. 4A). SR-4784 induced cyclin K degradation after 120 minutes of incubation, although requiring higher concentrations than SR-4835 (Fig. 4B), and showed reduced cell viability in wild type cells compared to DDB1-knockdown cells (Fig. S7, ESI†). SR-4784-PAP also degraded cyclin K in a proteasome-dependent manner, an effect diminished in DDB1-knockdown cells (Fig. 4C). For the drug-pulldown studies, cells treated with SR-4784-PAP (2 μM for 2 hours) were exposed to UV radiation (365 nm) to crosslink drug-target protein conjugates. Subsequently, the alkyne handle of SR-4784-PAP was biotinylated using click chemistry, and the biotinylated drug-target protein conjugates were enriched with neutravidin beads. Neutravidin pull-down assays revealed that DDB1 was enriched in the drug–protein conjugates with SR-4784-PAP compared to SR-4784 alone (Fig. 4D and E).

### *De novo* design of cyclin K degraders

**Fig. 5:**
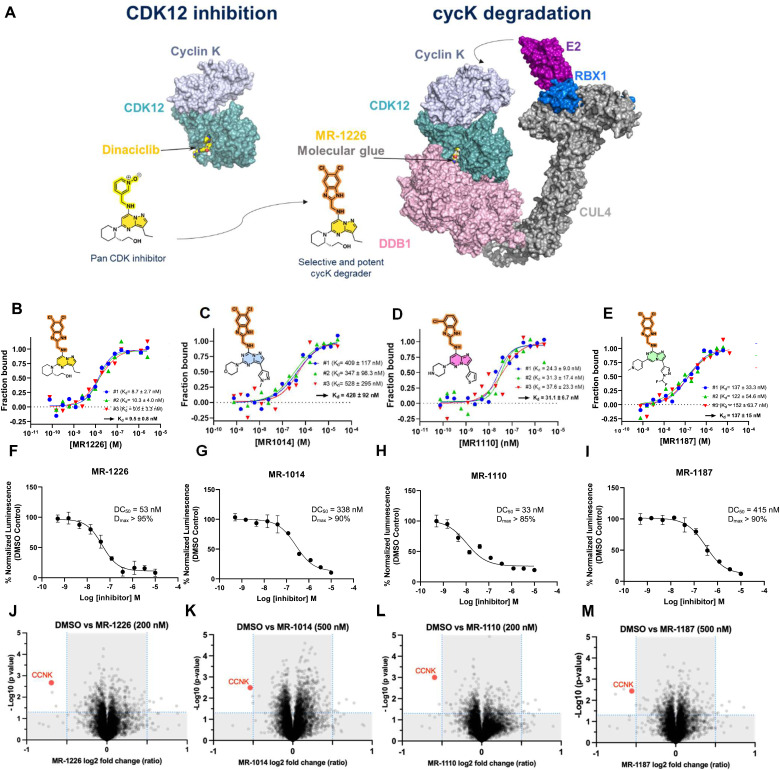
Cyclin K degraders by design. (A) *De novo* design of cyclin K molecular glue degraders from CDK12/pan-CDK inhibitors (dinaciclib). (B)–(E) Microscale thermophoresis studies assessing CDK12/cyclin K + DDB1 ternary complex formation induced by compound of interest. (F)–(I) Degradation of HiBiT tagged cyclin K using with MR-1187, MR-1110, MR-1014 and MR-1226 (A549 cell lines). (J)–(M) Label-free TMT-based quantitative proteomics upon treatment with MR-1187 (500 nM), MR-1110 (200 nM), MR-1014 (500 nM), MR-1226 (200 nM) at 2 h. Plot shows the significantly differentially abundant proteins ranked according to their *p* value (*Y*-axis) as −log_10_ and their relative abundance log_2_ ratio (*X* axis) between DMSO control and the compound of interest. All samples were generated in triplicates and 0.5 fold changes cut off were used for avg log_2_ ratio's to analyze the data. The *p* values < 0.05 were considered as significant.

SR-4835 occupies the ATP-binding pocket of CDK12 and bridges the CDK12–DDB1 interface through its 5,6-dichloro benzimidazole ring recruiting the DDB1–CUL4–RBX1 E3 ligase core to ubiquitinate cyclin K. These findings, supported by extensive structure–activity relationship (SAR) and structure-degradation relationship (SDR) data, indicate that the presence of a benzimidazole moiety confers gain-of-function activity to 2,6-disubstituted-*N*9-(heteroaryl)purines, resulting in cyclin K degradation and consequent kinase inactivation. Encouraged by these data, we hypothesized that the substituted benzimidazole moiety could be appended onto other CDK12/13 targeting ligands to induce cyclin K degradation. Thus, we appended this motif onto a structurally similar CDK12/pan-CDK inhibitor, dinaciclib, to generate compound MR-1226 (Fig. 5A). MR-1226 effectively degraded cyclin K with a DC_50_ of 50 nM (Fig. 5F), demonstrating that this chemical motif is versatile and can be adapted to other similar ligands to promote targeted cyclin K degradation.

Expanding on this approach, we attached the benzimidazole moiety to three different types of purine bioisosteres, resulting in the development of novel cyclin K degraders (Fig. 5B–M and Fig. S8 and S9, ESI†). These included pyrazolo[1,5-*a*][1,3,5]triazine (MR-1014), imidazo[1,2-*b*]pyridazine (MR-1110), and imidazo[1,2-*a*]pyrazine (MR-1187) series of analogs, demonstrating that diverse binder scaffolds can effectively trigger degradation when solvent-exposed groups are properly aligned to mediate interactions between CDK12 and DDB1. Each parent compound displayed kinase inhibition in CDK12, with MR-1226 showing a 35-fold better inhibition compared to the imidazo[1,2-*a*]pyrazine (MR-1187) analog, which in turn showed a smaller, 6-fold improvement in degradation efficiency as measured by DC_50_. Interestingly, although the imidazo[1,2-*a*]pyrazine-based compounds were generally the least active degraders, their weaker CDK12 functional activity did not consistently affect degradation affinity.

MST-based SAR studies revealed the varying effectiveness of cyclin K degraders in promoting the ternary complex, MR-1226 and MR-1014 being the most (*K*_d_ = 9.5 nM) and least potent (*K*_d_ = 428 nM) compounds, respectively (Fig. 5B–E). Pearson analysis revealed a significant correlation between ternary complex formation and cyclin K degradation (*P* = 0.026), whereas the inhibition of CDK12/cycK enzymatic activity showed a trend but lacked statistical significance (*P* = 0.053) (Fig. S10, ESI†). The data indicate that the ability of a molecular glue to induce the ternary complex correlates with cyclin K degradation rather than the inhibition of CDK12/cycK enzymatic activity.

To assess proteome-wide degradation selectivity, we conducted proteomics studies. Quantitative proteome-wide mass spectrometry was performed on MDA-MB-231 cells treated with each of the four designed compounds (plus SR-4835, Fig. S11, ESI†). Despite variations in the degradation profiles among the compounds, cyclin K consistently emerged as one of the most degraded proteins after a 2-hour treatment across all samples (Fig. 5J–M).

Collectively our findings demonstrate that modifications to the core structure with a benzimidazole side chain in the solvent-exposed region can effectively mediate the interaction between CDK12 and DDB1, enabling the successful development of cyclin K degraders through *de novo* design.

## Discussion

Our study highlights the successful discovery and design of molecular glue enhancers that facilitate CDK12–DDB1 interactions, leading to the targeted degradation of cyclin K. While Kozicka *et al.*^29^ identified multiple published kinase inhibitors, including SR-4835 as cryptic degraders of cyclin K, our work further clarifies and reinforces the understanding of cyclin K degraders operating *via* DDB1 recruitment. The CDK12 inhibitor SR-4835 facilitates potent molecular glue activity enabling the degradation of cyclin K through the formation of a ternary complex involving CDK12, DDB1, and the small molecule itself. The degradation was shown to correlate with the compound's enhanced ability to inhibit cell growth, underscoring the therapeutic potential of this approach in cancer treatment.^22^

The biochemical characterization of SR-4835 revealed its ability to induce interactions between CDK12/CycK and DDB1, as confirmed through various assays, including size-exclusion chromatography, dynamic light scattering, differential scanning fluorimetry, microscale thermophoresis and pulldown experiments. These assays collectively demonstrated that SR-4835 promotes the formation of higher molecular weight ternary complexes and stabilizes the heterodimeric CDK12/CycK complex in the presence of DDB1. Our results also indicate that the molecular glue activity of SR-4835 is mechanistically dependent on the functionality of the CUL4–RBX1–DDB1 ubiquitin ligase. Knockdown experiments with DDB1 and other components of the ubiquitin-proteasome system confirmed that cyclin K degradation induced by SR-4835 is mediated through this pathway. Furthermore, the capacity of SR-4835 to induce the formation of a ternary complex between CDK12, DDB1, and cyclin K was shown to be a critical determinant of its efficacy as a cyclin K degrader.

A key finding of our study is the elucidation of the crystal structure of the ternary DDB1ΔB·SR-4835·CDK12/CycK complex. This structure provided critical insights into the molecular interactions that facilitate the formation of the ternary complex, enabling us to design molecular glues that convert various CDK12 scaffold inhibitors into effective cyclin K degraders. Notably, we demonstrated that the introduction of a surface-exposed benzimidazole moiety could strategically convert the clinical pan-CDK inhibitor dinaciclib into a potent cyclin K degrader, MR-1226, with a DC_50_ of 50 nM (*D*_max_ > 95%). In exploring the potential of various chemical modifications to enhance the molecular glue activity, we found that appending a benzimidazole moiety to different pan-CDK/CDK12 inhibitors significantly improved their ability to degrade cyclin K. This was evident in the successful design of novel cyclin K degraders such as MR-1014, MR-1110, and MR-1187, each demonstrating varying degrees of effectiveness in promoting ternary complex formation and cyclin K degradation. In a parallel experiment, we explored whether appending the phenylpyridine ring system from CR8 to in-house inhibitors could drive the interaction of CDK12 with DDB1 (Fig. S12, ESI†). However, this modification did not yield active compounds, highlighting the versatility and efficacy of the substituted benzimidazole moiety as a chemical handle for converting CDK12 inhibitors into cyclin K degraders.

The data suggest that the capacity of compounds to induce a ternary complex correlates with the degradation of cyclin K but not necessarily with the inhibition of CDK12/cyclin K enzymatic activity. This insight is crucial for the future design of molecular glues, as it emphasizes the importance of ternary complex formation in achieving targeted protein degradation.

Overall, our findings underscore significant progress in the rational design of molecular glues for targeted protein degradation. The ability to convert existing kinase inhibitors into degraders of key regulatory proteins like cyclin K opens new avenues for therapeutic intervention in cancers driven by dysregulated CDK12 activity. Future studies will focus on optimizing these molecular glue degraders for clinical applications and exploring their potential in combination with other cancer therapies.

## Methods

### Small molecule inhibitors

(A) Cayman Chemical – proteasomal inhibitor (*S*)-MG132 (NC0022110).

(B) Med Chem Express – (*R*)-CR8 (HY-18340), THZ531 (HY-103618), pevonidistat (HY-70062), TAK243 (HY-100487).

### Synthesis

All reagents were purchased from commercial suppliers and were used without further purification. Dichloromethane, ethanol, 1,4-dioxane, diethyl ether, *N*,*N*-dimethylformamide, acetic acid and ethyl acetate were dried by being passed through a column of desiccant (activated A-1 alumina). *N*-Bromo succinimide, sodium ethoxide (21% w/w in ethanol) and LiHMDS (1 M in THF) was purchased from Sigma-Aldrich and used as such. Reactions were either monitored by thin-layer chromatography or analytical LC-MS. Thin layer chromatography was performed on Kieselgel 60 F254 glass plates pre-coated with a 0.25 mm thickness of silica gel. TLC plates were visualized with UV light and/or by staining with ninhydrin solution. Normal phase column chromatography was performed on a Biotage Selekt automated flash system. Compounds were loaded onto pre-filled cartridges filled with KP-Sil 50 μm irregular silica. For microwave reactions, a Biotage Initiator Microwave system was used. Final products were isolated by reverse-phase HPLC using Waters HPLC system with UV detector, with Atlantis T3 OBD Prep Column, 100 Å, 5 μm, 19 mm × 150 mm. Compounds were eluted using a gradient elution of 90/10 to 0/100 A/B over 20 min at a flow rate of 20.0 mL min^−1^, where solvent A was water (+0.1% formic acid), and solvent B was acetonitrile.

The structures of all compounds were verified *via*^1^H NMR, ^19^F NMR and LCMS. The purity of isolated products was determined using an LC-MS instrument (Agilent 1290 Infinity series LC with single quadrupole MSD system, AP-ESI Ion Source) equipped with Kinetex® 1.7 μm C18 100 Å, LC column 50 × 2.1 mm, Ea (Phenomenex) column. Elution was performed using the following conditions: 2% (v/v) acetonitrile (+0.1% FA) in 98% (v/v) H_2_O (+0.1% FA), ramped to 98% acetonitrile over 4.0 min, and holding at 98% acetonitrile for 0.5 min with a flow rate of 0.6 mL min^−1^; UV absorption was detected from 200 to 950 nm using a diode array detector. The purity of each compound was ≥95% based on this analysis.

NMR spectra were recorded at ambient temperature on a 500 MHz Bruker NMR spectrometer in DMSO-d_6_. All ^1^H NMR data are reported in parts per million (ppm) downfield of TMS and were measured relative to the signals for dimethyl sulfoxide (2.50 ppm). ^19^F NMR experiments were performed with ^1^H decoupling. Data for H NMR are reported as follows: chemical shift (*δ*, ppm), multiplicity (s = singlet, d = doublet, t = triplet, q = quartet, m = multiplet), integration, and coupling constant (Hz). NMR data was analyzed and processed by using MestReNova software.
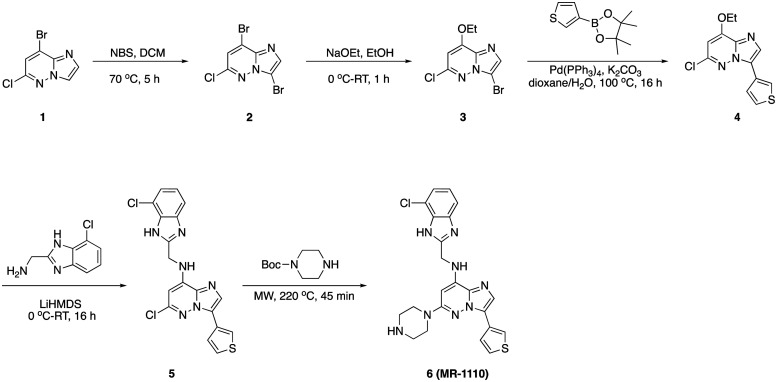


### 3,8-Dibromo-6-chloroimidazo[1,2-*b*]pyridazine (2)

A solution of 8-bromo-6-chloroimidazo[1,2-*b*]pyridazine, 1 (10.0 g, 43.02 mmol) and *N*-bromo succinimide (8.42 g, 47.32 mmol) in dichloromethane (80 mL) was heated to 70 °C in a sealed tube. After completion, solvent was evaporated and water was added to the residue resulting in an off-white precipitate which was filtered and dried to afford 3,8-dibromo-6-chloroimidazo[1,2-*b*]pyridazine, 2 (12.0 g, 89.59%) as an off-white solid. ^1^H NMR (500 MHz, DMSO) *δ* 8.21 (d, *J* = 10.5 Hz, 1H), 7.82 (d, *J* = 6.1 Hz, 1H). LCMS (*m*/*z*): 311.8 (M + H)^+^, *R*_t_: 2.577 min.

### 3-Bromo-6-chloro-8-ethoxyimidazo[1,2-*b*]pyridazine (3)

To a solution of 3,8-dibromo-6-chloroimidazo[1,2-*b*]pyridazine, 2 (12.0 g, 38.54 mmol) in ethanol (50 mL) at 0 °C was added sodium ethanolate (28.0 mL, 77.08 mmol) dropwise and stirred at room temperature for 1 h. After completion, solvent was evaporated. The residue was treated with 1 N NH_4_Cl in water and extracted with dichloromethane (3 × 50 mL). The combined organic layers were concentrated, and the residue was purified by column chromatography on silica gel (20–30% ethyl acetate in hexanes) to afford 3-bromo-6-chloro-8-ethoxyimidazo[1,2-*b*]pyridazine, 3 (8.0 g, 75.07%) as a pale-orange solid. ^1^H NMR (500 MHz, DMSO) *δ* 7.79 (s, 1H), 7.00 (s, 1H), 4.40 (q, *J* = 7.0 Hz, 2H), 1.44 (t, *J* = 7.0 Hz, 3H). LCMS (*m*/*z*): 277.9 (M + H)^+^, *R*_t_: 2.523 min.

### 6-Chloro-8-ethoxy-3-(thiophen-3-yl)imidazo[1,2-*b*]pyridazine (4)

Argon gas was bubbled to a mixture of 3-bromo-6-chloro-8-ethoxyimidazo[1,2-*b*]pyridazine, 3 (1.0 g, 3.62 mmol), thiophen-3-ylboronic acid (509 mg, 3.98 mmol) and potassium carbonate (1.0 g, 7.23 mmol) in 1,4-dioxane (20 mL) and water (4 mL) for 10 min. To this mixture, Pd(PPh_3_)_4_ (417 mg, 0.1 mmol) was added, and the reaction was heated to 90 °C for 16 h. After completion, the reaction was cooled to room temperature and was transferred to separatory funnel containing brine (50 mL) and the aqueous layer was extracted with ethyl acetate (2 × 50 mL). The combined organic layers were dried over sodium sulphate and concentrated under reduced pressure. The residue was purified by column chromatography on silica gel (20–50% ethyl acetate in hexanes) to afford 6-chloro-8-ethoxy-3-(thiophen-3-yl)imidazo[1,2-*b*]pyridazine, 4 (0.8 g, 79.08%) as an off-white solid. ^1^H NMR (500 MHz, DMSO) *δ* 8.29 (dd, *J* = 2.9, 1.3 Hz, 1H), 8.14 (s, 1H), 7.79 (dd, *J* = 5.1, 1.3 Hz, 1H), 7.73 (dd, *J* = 5.1, 2.9 Hz, 1H), 6.99 (s, 1H), 4.43 (q, *J* = 7.0 Hz, 2H), 1.46 (t, *J* = 7.0 Hz, 3H). LCMS (*m*/*z*): 280.0 (M + H)^+^, *R*_t_: 2.969 min.

### 6-Chloro-*N*-((7-chloro-1*H*-benzo[*d*]imidazol-2-yl)methyl)-3-(thiophen-3-yl)imidazo[1,2-*b*]pyridazin-8-amine (5)

To a solution of 6-chloro-8-ethoxy-3-(thiophen-3-yl)imidazo[1,2-*b*]pyridazine, 4 (200 mg, 0.715 mmol) and benzimidazole (155 mg, 0.715 mmol) at 0 °C under argon atm was added LiHMDS (2.86 mL, 2.86 mmol, 1 M in THF) dropwise. The reaction turned to dark brown. The solution was allowed to warm to room temperature and stirred for 16 h. After completion, the reaction was quenched with brine (30 mL) and the aqueous layer was extracted with ethyl acetate (2 × 30 mL). The combined organic layers were dried over sodium sulphate and concentrated under reduced pressure. The residue was purified by column chromatography on silica gel (2–5% methanol in dichloromethane) to afford 6-chloro-*N*-((7-chloro-1*H*-benzo[*d*]imidazol-2-yl)methyl)-3-(thiophen-3-yl)imidazo[1,2-*b*]pyridazin-8-amine, 5 (0.2 g, 62.20%) as an off-white solid. ^1^H NMR (500 MHz, DMSO) *δ* 12.76 (s, 1H), 8.44 (d, *J* = 18.9 Hz, 1H), 8.27 (dd, *J* = 3.0, 1.3 Hz, 1H), 8.06 (s, 1H), 7.77 (dd, *J* = 5.1, 1.3 Hz, 1H), 7.71 (dd, *J* = 5.1, 2.9 Hz, 1H), 7.50–7.40 (m, 1H), 7.24 (d, *J* = 7.6 Hz, 1H), 7.16 (t, *J* = 7.9 Hz, 1H), 6.40 (s, 1H), 4.98–4.78 (m, 2H). LCMS (*m*/*z*): 416.0 (M + H)^+^, *R*_t_: 3.380 min.

### *N*-((7-Chloro-1*H*-benzo[*d*]imidazol-2-yl)methyl)-6-(piperazin-1-yl)-3-(thiophen-3-yl)imidazo[1,2-*b*]pyridazin-8-amine (6)

To a flame-dry microwave vial, (50 mg, 0.011 mmol) of 6-chloro-*N*-((7-chloro-1*H*-benzo[*d*]imidazol-2-yl)methyl)-3-(thiophen-3-yl)imidazo[1,2-*b*]pyridazin-8-amine, 5 in 1,4-dioxane (1 mL) was added *tert*-butyl piperazine-1-carboxylate (103 mg, 0.055 mmol). The reaction mixture was heated to 220 °C in a microwave for 45 min. After completion, solvent was removed under reduced pressure and purified by PREP HPLC purification, where solvent A was water (+0.1% formic acid), and solvent B was acetonitrile. Pure fraction was concentrated under reduced pressure and the residue was triturated with diethyl ether and dried in high vacuo to afford *N*-((7-chloro-1*H*-benzo[*d*]imidazol-2-yl)methyl)-6-(piperazin-1-yl)-3-(thiophen-3-yl)imidazo[1,2-*b*]pyridazin-8-amine, 6 (24 mg, 71.63%) as pale-yellow solid. ^1^H NMR (500 MHz, DMSO) *δ* 12.70 (s, 1H), 8.28 (dd, *J* = 3.0, 1.2 Hz, 1H), 8.21 (s, 1H), 7.84 (s, 1H), 7.74 (dd, *J* = 5.1, 1.2 Hz, 1H), 7.65 (dd, *J* = 5.1, 3.0 Hz, 2H), 7.44 (d, *J* = 7.8 Hz, 1H), 7.23 (d, *J* = 7.7 Hz, 1H), 7.15 (t, *J* = 7.8 Hz, 1H), 6.15 (s, 1H), 4.83 (d, *J* = 6.1 Hz, 2H), 3.46–3.40 (m, 4H), 2.97–2.86 (m, 4H). LCMS (*m*/*z*): 465.1 (M + H)^+^, *R*_t_: 1.990 min.
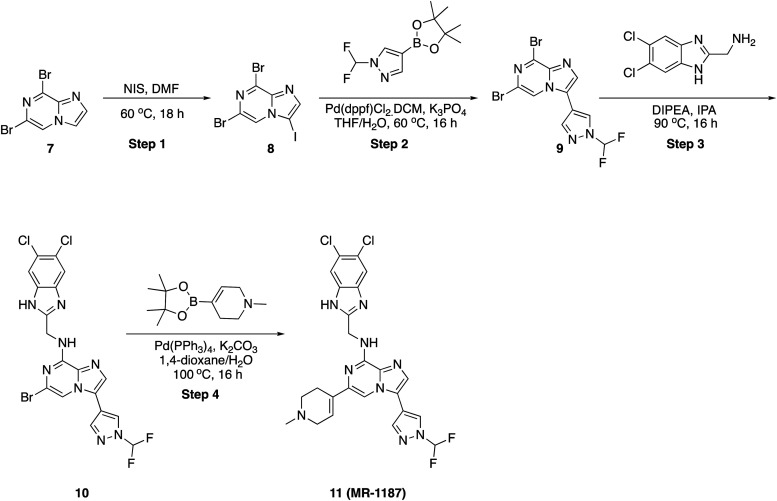


### 6,8-Dibromo-3-iodoimidazo[1,2-*a*]pyrazine (8)

A solution of 6,8-dibromoimidazo[1,2-*a*]pyrazine, 7 (5.0 g, 18.06 mmol) and *N*-iodo succinimide (4.27 g, 18.96 mmol) in *N*,*N*-dimethyl formamide (50 mL) was heated to 60 °C in a sealed tube for 16 h. After completion, solvent was evaporated *in vacuo*, diluted with ethyl acetate (100 mL), and washed with aqueous saturated sodium thiosulphate (3 × 100 mL) and, the organic layer was dried over sodium sulphate and concentrated and dried *in vacuo* to afford 6,8-dibromo-3-iodoimidazo[1,2-*a*]pyrazine, 8 (5.50 g, 75.62%) as an off-white solid. ^1^H NMR (500 MHz, DMSO) *δ* 8.65 (s, 1H), 8.03 (s, 1H). LCMS (*m*/*z*): 403.7 (M + H)^+^, *R*_t_: 2.452 min.

### 6,8-Dibromo-3-(1-(difluoromethyl)-1*H*-pyrazol-4-yl)imidazo[1,2-*a*]pyrazine (9)

A solution of 6,8-dibromo-3-iodoimidazo[1,2-*a*]pyrazine, 8 (200 mg, 0.496 mmol) in dioxane (8 mL) and water (2 mL) were added 1-difluoro methyl-4-(4,4,5,5-tetramethyl-1,3,2-dioxaborolan-2-yl)-1*H*-pyrazole (121 mg, 0.496 mmol) and potassium carbonate (103 mg, 0.745 mmol) at RT. Then the reaction mixture was purged with nitrogen for 10 min, followed by the addition of Pd(PPh_3_)_4_ (29 mg, 5 mol%). Then the reaction mixture was stirred at 90 °C for 16 h. After completion, the reaction mixture was diluted with water (10 mL) and, extracted with ethyl acetate (2 × 20 mL) and dried over sodium sulphate. The crude compound was purified by column over silica gel 230–400 mesh, product eluted at 60% ethyl acetate in hexane to afford the pure product (140 mg, 71.75%) as an off-white solid. ^1^H NMR (500 MHz, DMSO) *δ* 8.90 (s, 1H), 8.52 (s, 1H), 8.35 (s, 1H), 8.11–7.87 (m, 2H), ^19^F NMR (471 MHz, DMSO) *δ* −94.57, −94.69. LCMS (*m*/*z*): 393.8 (M + H)^+^, *R*_t_: 2.456 min.

### 6-Bromo-*N*-((5,6-dichloro-1*H*-benzo[*d*]imidazol-2-yl)methyl)-3-(1-(difluoromethyl)-1*H*-pyrazol-4-yl)imidazo[1,2-*a*]pyrazin-8-amine (10)

To a stirred solution of 6-bromo-*N*-((5,6-dichloro-1*H*-benzo[*d*]imidazol-2-yl)methyl)-3-(1-(difluoromethyl)-1*H*-pyrazol-4-yl)imidazo[1,2-*a*]pyrazin-8-amine, 9 (120 mg, 0.305 mmol) and (5,6-dichloro-1*H*-benzo[*d*]imidazol-2-yl)methanamine (79 mg, 0.366 mmol) in 2-propanol (2 mL) was added *N*,*N*-diisopropyl ethylamine (59 mg, 0.458 mmol) in dropwise. Then, the reaction mixture was heated at 90 °C for 16 h. After completion, the reaction mixture was concentrated to dryness, diluted with water (20 mL) and extracted with ethyl acetate (2 × 20 mL). The combined organic layers were dried over sodium sulphate and concentrated under reduced pressure. The residue was purified by column chromatography on silica gel (2–5% methanol in dichloromethane) to afford the pure product (120 mg, 74.41%) as an off-white solid. ^1^H NMR (500 MHz, DMSO) *δ* 8.81 (s, 1H), 8.45 (s, 2H), 8.09–7.96 (m, 2H), 7.73 (s, 2H), 6.38 (s, 1H), 4.84 (s, 2H), ^19^F NMR (471 MHz, DMSO) *δ* −94.35, −94.48. LCMS (*m*/*z*): 528.9 (M + H)^+^, *R*_t_: 2.890 min.

### *N*-((5,6-Dichloro-1*H*-benzo[*d*]imidazol-2-yl)methyl)-3-(1-(difluoromethyl)-1*H*-pyrazol-4-yl)-6-(1-methyl-1,2,3,6-tetrahydropyridin-4-yl)imidazo[1,2-*a*]pyrazin-8-amine (11)

To a stirred solution of 6-bromo-*N*-((5,6-dichloro-1*H*-benzo[*d*]imidazol-2-yl)methyl)-3-(1-(difluoromethyl)-1*H*-pyrazol-4-yl)imidazo[1,2-*a*]pyrazin-8-amine, 10 (50 mg, 0.094 mmol) in dioxane (1 mL) and water (0.3 mL) was added 1-methyl-4-(4,4,5,5-tetramethyl-1,3,2-dioxaborolan-2-yl)-1,2,3,6-tetrahydropyridine (32 mg, 0.142 mmol) and K_2_CO_3_ (40 mg, 0.284 mmol), then the reaction mixture was purged with argon for 10 min, Pd(PPh_3_)_4_ (11 mg, 10 mol%) was added and stirred at 100 °C for 16 h. After completion, solvent was removed under reduced pressure and purified by PREP HPLC purification, where solvent A was water (+0.1% formic acid), and solvent B was acetonitrile. Pure fraction was concentrated under reduced pressure and the residue was triturated with diethyl ether and dried in high vacuo. Yield (18 mg, 34.93%), pale-yellow solid. ^1^H NMR (500 MHz, DMSO) *δ* 12.55 (s, 1H), 8.86 (s, 1H), 8.33 (s, 1H), 8.26 (s, 1H), 8.14 (t, *J* = 5.8 Hz, 1H), 7.90 (s, 1H), 7.80 (s, 1H), 7.54 (s, 1H), 6.55 (d, *J* = 3.6 Hz, 1H), 4.92 (d, *J* = 5.8 Hz, 2H), 2.94 (q, *J* = 2.9 Hz, 2H), 2.43 (d, *J* = 5.6 Hz, 2H), 2.24 (s, 3H), ^19^F NMR (471 MHz, DMSO) *δ* −93.96 to −94.51 (m), LCMS (*m*/*z*): 545.1 (M + H)^+^, *R*_t_: 2.117 min.
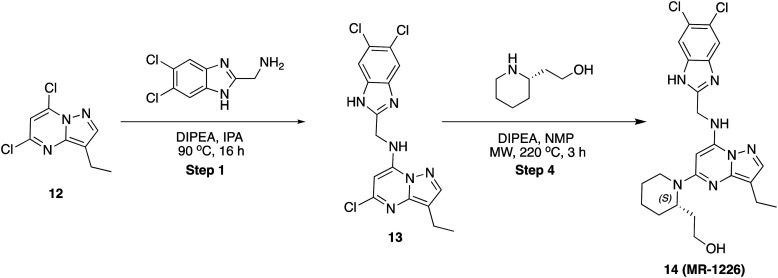


### 5-Chloro-*N*-((5,6-dichloro-1*H*-benzo[*d*]imidazol-2-yl)methyl)-3-ethylpyrazolo[1,5-*a*]pyrimidin-7-amine (13)

To a stirred solution of 5,7-dichloro-3-ethylpyrazolo[1,5-*a*]pyrimidine, 12 (80 mg, 0.37 mmol) and (5,6-dichloro-1*H*-benzo[*d*]imidazol-2-yl)methanamine (80 mg, 0.370 mmol) in 2-propanol (2 mL) was added *N*,*N*-diisopropyl ethylamine (96 mg, 0.74 mmol) in dropwise. Then, the reaction mixture was heated at 90 °C for 16 h. After completion, the reaction mixture was concentrated to dryness, diluted with water (20 mL) and extracted with ethyl acetate (2 × 20 mL). The combined organic layers were dried over sodium sulphate and concentrated under reduced pressure. The residue was purified by column chromatography on silica gel (2–5% methanol in dichloromethane) to afford the pure product (80 mg, 54.61%) as an off-white solid. LCMS (*m*/*z*): 397.0 (M + 2H)^+^, *R*_t_: 3.030 min.

### (*S*)-2-(1-(7-(((5,6-Dichloro-1*H*-benzo[*d*]imidazol-2-yl)methyl)amino)-3-ethylpyrazolo[1,5-*a*]pyrimidin-5-yl)piperidin-2-yl)ethan-1-ol (14)

To a flame-dry microwave vial, (40 mg, 0.101 mmol) of 5-chloro-*N*-((5,6-dichloro-1*H*-benzo[*d*]imidazol-2-yl)methyl)-3-ethylpyrazolo[1,5-*a*]pyrimidin-7-amine, 13 in *N*-methyl pyrrolidine (1 mL) was added (*S*)-2-(piperidin-2-yl)ethan-1-ol (65 mg, 0.505 mmol) and *N*,*N*-diisopropyl ethylamine (65 mg, 0.505 mmol). The reaction mixture was heated to 220 °C in a microwave for 3 h. After completion, solvent was removed under reduced pressure and purified by PREP HPLC purification, where solvent A was water (+0.1% formic acid), and solvent B was acetonitrile. Pure fraction was concentrated under reduced pressure and the residue was triturated with diethyl ether and dried in high vacuo. Yield (12 mg, 24.30%), pale-yellow solid. ^1^H NMR (500 MHz, DMSO) *δ* 8.38 (s, 1H), 7.78 (s, 2H), 7.69 (s, 1H), 5.66 (s, 1H), 4.79 (dd, *J* = 6.3, 2.2 Hz, 2H), 4.58 (s, 1H), 4.21 (s, 1H), 3.54–3.46 (m, 2H), 3.36 (dd, *J* = 18.5, 6.4 Hz, 4H), 3.11–3.06 (m, 1H), 2.86–2.76 (m, 2H), 1.64–1.58 (m, 4H), 1.56–1.52 (m, 2H), 1.18 (t, *J* = 7.5 Hz, 3H). LCMS (*m*/*z*): 488.2 (M + H)^+^, *R*_t_: 4.793 min.
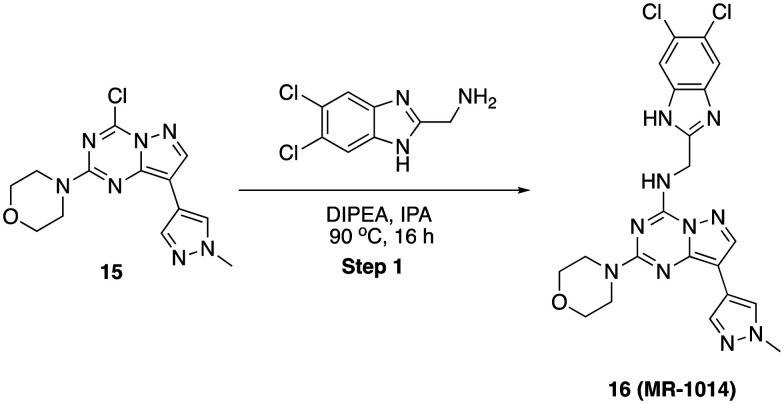


### *N*-((5,6-Dichloro-1*H*-benzo[*d*]imidazol-2-yl)methyl)-8-(1-methyl-1*H*-pyrazol-4-yl)-2-morpholinopyrazolo[1,5-*a*][1,3,5]triazin-4-amine (16)

To a stirred solution of 4-(4-chloro-8-(1-methyl-1*H*-pyrazol-4-yl)pyrazolo[1,5-*a*][1,3,5]triazin-2-yl)morpholine, 15 (40 mg, 0.125 mmol) and (5,6-dichloro-1*H*-benzo[*d*]imidazol-2-yl)methanamine (30 mg, 0.137 mmol) in 2-propanol (2 mL) was added *N*,*N*-diisopropyl ethylamine (63 mg, 0.625 mmol) in dropwise. Then, the reaction mixture was heated at 90 °C for 16 h. After completion, solvent was removed under reduced pressure and purified by PREP HPLC purification, where solvent A was water (+0.1% formic acid), and solvent B was acetonitrile. Pure fraction was concentrated under reduced pressure and the residue was triturated with diethyl ether and dried in high vacuo. Yield (16 mg, 25.61%), white solid. ^1^H NMR (500 MHz, DMSO) *δ* 12.57 (s, 1H), 8.95 (s, 1H), 8.27 (s, 1H), 8.13 (s, 1H), 7.92 (s, 1H), 7.75 (s, 1H), 6.55 (s, 1H), 4.81 (d, *J* = 4.1 Hz, 2H), 3.79 (s, 3H), 3.58 (s, 4H), 3.49–3.43 (m, 4H). LCMS (*m*/*z*): 499.0 (M + H)^+^, *R*_t_: 2.659 min.
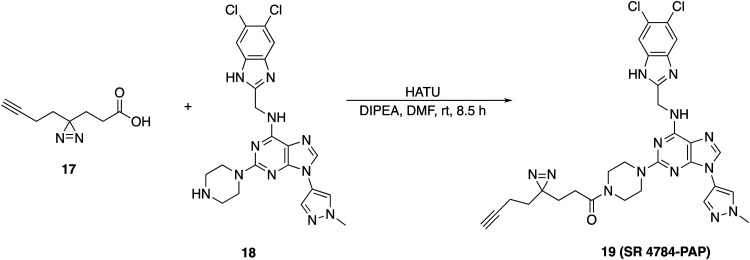


### 3-(3-(But-3-yn-1-yl)-3*H*-diazirin-3-yl)-1-(4-(6-(((5,6-dichloro-1*H*-benzo[*d*]imidazol-2-yl)methyl)amino)-9-(1-methyl-1*H*-pyrazol-4-yl)-9*H*-purin-2-yl)piperazin-1-yl)propan-1-one (19)

To a solution of 3-(3-(but-3-yn-1-yl)-3*H*-diazirin-3-yl)propanoic acid, 17 (0.012 g, 0.073 mmol) that was synthesized according to the reported procedure ACS Chem. Biol., 2023, **18**(2), 251–264 in DMF (1.0 mL) was added HATU (0.035 g, 0.092 mmol) and DIPEA (0.052 mL, 0.294 mmol). The reaction mixture was stirred for 15 min, at this time *N*-((5,6-dichloro-1*H*-benzo[*d*]imidazol-2-yl)methyl)-9-(1-methyl-1*H*-pyrazol-4-yl)-2-(piperazin-1-yl)-9*H*-purin-6-amine, 18 (0.043 g, 0.081 mmol) was added and stirred at rt for 8.5 h. The reaction mixture was diluted with ethyl acetate, washed with water and brine then dried over sodium sulfate. The organics were removed under reduced pressure to give crude 19. The residue was purified *via* PREP-HPLC (solvent A: +0.1% formic acid in water, solvent B: acetonitrile) to afford 3-(3-(but-3-yn-1-yl)-3*H*-diazirin-3-yl)-1-(4-(6-(((5,6-dichloro-1*H*-benzo[*d*]imidazol-2-yl)methyl)amino)-9-(1-methyl-1*H*-pyrazol-4-yl)-9*H*-purin-2-yl)piperazin-1-yl)propan-1-one, 19 as an off-white solid (0.028 g, 59%). ^1^H NMR (500 MHz, DMSO) *δ* 12.46 (s, 1H), 8.31 (s, 1H), 8.21 (s, 1H), 7.99 (s, 1H), 7.72 (s, 2H), 4.82 (s, 2H), 3.92 (s, 3H), 3.58 (d, *J* = 25.6 Hz, 4H), 3.34 (s, 4H), 2.82 (t, *J* = 2.6 Hz, 1H), 2.14 (t, *J* = 7.7 Hz, 2H), 1.99 (td, *J* = 7.4, 2.7 Hz, 2H), 1.61 (dt, *J* = 14.5, 7.5 Hz, 4H). LCMS (*m*/*z*): 647.1 (M + H)^+^, *R*_t_: 2.737 min.

### Cell-culture

MDA-MB-231 (human triple-negative breast cancer cells) and HEK293T (human embryonic kidney cells) were purchased from the American type culture collection (ATCC) and maintained in Dulbecco's modified Eagle's medium (DMEM) (Gibco) supplemented with 10% (v/v) heat-inactivated fetal bovine serum (FBS) (Gibco) and penicillin/streptomycin (100 U mL^−1^ each). HiBiT-CCNK KI A549 cells, purchased from Promega, were maintained in F12 medium supplemented with 10% (v/v) heat-inactivated fetal bovine serum (FBS) (Gibco) and penicillin/streptomycin (100 U mL^−1^ each). DDB1-knockdown MDA-MB-231 and DDB1-knockdown HEK293T cell lines were maintained in the same media formulation as their corresponding wild-type cell lines. All cell lines were cultured at 37 °C in an incubator with CO_2_ levels maintained at 5%.

### Antibodies

The antibodies used in this study were as follows: anti-cyclin K (rabbit, Bethyl Labs; rabbit, Thermo Fisher), anti-DDB1 (rabbit, Cell Signaling Technology; mouse, Santa Cruz Biotechnology), anti-CDK12 (rabbit, Abcam; rabbit, Cell Signaling Technology), anti-Cullin 4 (rabbit, Cell Signaling Technology), anti-Cas9 (mouse, Cell Signaling Technology), and anti-GAPDH (mouse, Sigma).

### Protein expression and purification (Moffitt)

The gene of human DDB1 (Uniprot: Q16531) with a beta-propeller B domain deletion (ΔBPB: a.a. 396–705 deleted) was subcloned into pFastBac1 vector with an N-terminal His_8_-tag and a HRV 3C cleavage site (GeneScript), and the recombinant protein was expressed in Spodoptera frugiperda SF9 insect cells in sf900™ II SFM culture medium using the Bac to Bac™ baculovirus expression system (Gibco™). Cell pellets were harvested 48 hours post infection *via* centrifugation and lysed in buffer A (50 mM Tris–HCl (pH 8.0), 200 mM NaCl, 10 mM imidazole, 0.5 mM tris(2-carboxyethyl) phosphine (TCEP)) supplemented with EDTA-free Pierce™ protease inhibitor tablet (Thermo Fisher Scientific). After removal of DNA *via* precipitation with 0.05% polyethyleneimine (PEI) 60 K (Acros), the lysate was cleared by centrifugation, loaded onto a 50 mL Superflow Ni-NTA resin (QIAGEN) affinity column, washed with buffer A, and eluted in buffer A including 500 mM imidazole. The peak fractions were combined, concentrated *via* Amicon ultra centrifugal filters (Millipore), and subjected to size-exclusion chromatography using a Superdex 200 26/60 column equilibrated with 50 mM HEPES (pH 7.4), 200 mM NaCl, 2.5% (v/v) glycerol and 0.25 mM TCEP. The concentrated protein at 2 mg mL^−1^ was flash-frozen in liquid nitrogen and stored at −80 °C.

The kinase domain of human CDK12 (Uniprot: Q9NYV4; a.a. 715–1050) with an N terminal GST tag and a tobacco etch virus (TEV) cleavage site was subcloned under the polyhedron promoter, and the cyclin box of human cyclin K (Uniprot: O75909; a.a. 11–267) was subcloned under the p10 promoter of the same pFastBac Dual vector for co-expression of both proteins. Full-length *S. cerevisiae* CAK1 (Uniprot: P43568) with an N-terminal His_6_ tag was subcloned into pFastBac 1 vector. To produce enzymatically active CDK12/cyclinK complex, SF9 cells in ESF921 media (expression systems) were co-infected with a 1 : 1 ratio of baculovirus generated for both CDK12/cyclinK and CAK1, then harvested 72 hours post infection *via* centrifugation and lysed in buffer B (50 mM HEPES pH7.5, 500 mM NaCl, 10 mM MgCl2, 10% glycerol, 0.5 mM TCEP) supplemented with EDTA free Pierce™ protease inhibitor tablet (Thermo Fisher Scientific). After removal of DNA *via* precipitation with 0.05% PEI, the soluble lysate was cleared by centrifugation, loaded onto a 50 mL Glutathione Sepharose 4B resin (Cytiva) affinity column, washed with buffer B, and eluted with buffer B including 15 mM glutathione. The peak fractions were combined, concentrated *via* Amicon ultra centrifugal filters (Millipore), passed over a desalting column equilibrated with buffer B, and subjected to TEV protease digestion (protease to protein ratio = 1 : 25) at 4 °C overnight, resulting in removal of the GST-affinity tag. The sample was concentrated and further purified by size-exclusion chromatography using a Superdex 75 26/60 column in 50 mM HEPES (pH 7.4), 200 mM NaCl, 2.5% (v/v) glycerol and 0.25 mM TCEP. The eluted CDK12/cyclin K complex was concentrated to 2 mg mL^−1^, flash-frozen in liquid nitrogen and stored at −80 °C.

### Protein expression (University of Bonn)

Recombinant Cdk12/CycK and DDB1ΔB proteins were expressed in *Sf9* insect cells using the MultiBac^Turbo^ system.^33^*Sf9* cell cultures were maintained in SF-900 serum-free medium (Invitrogen) at 27 °C with agitation at 80 rpm. *E. coli* (DH10) MultiBac^Turbo^ cells were transformed with respective plasmids for integration into the baculoviral genome. Recombinant bacmid DNA was extracted and utilized for subsequent transfection of *Sf9* cells, followed by baculovirus amplification. For recombinant protein expression, a suspension culture of *Sf9* insect cells was infected at a density of 1.5 × 10^6^ cells per mL by adding 3% (v/v) baculovirus V2 preparation. After 72 h, cells were collected by centrifugation at 2000 rpm and 4 °C for 20 min in an Avanti J-26S XP centrifuge equipped with a JLA-8.1000 rotor (Beckman Coulter), washed with PBS, snap frozen in liquid nitrogen, and stored at −80 °C.

### Recombinant protein purification

Synthetic genes, codon optimized for expression in *Sf9* insect cells, comprising the kinase domain of human Cdk12 (residues 714–1063, UniProt accession number Q9NYV4) and cyclin box domains of human cyclin K (residues 1–267, UniProt accession number O75909) were purchased from GeneArt. The coding sequence of Cdk12 was integrated into the pACEBac1 acceptor vector, while CycK was introduced into pIDK donor vector. Both target vectors underwent modification, wherein a GST-affinity tag followed by a tobacco etch virus (TEV) protease cleavage site was introduced upstream of the protein coding sequence. A pIDC donor vector was employed to insert full-length CDK-activating kinase CAK1 (UniProt accession number P43568) from *S. cerevisiae* devoid of any affinity tag. Cre recombination was performed to combine all three vectors *in vitro*, resulting in fused vectors suitable for co-expression in *Sf9* insect cells. For purification of the Cdk12/CycK heterodimeric complex, a combination of sequential GST-affinity chromatography, TEV-mediated affinity tag removal, and size-exclusion chromatography was employed as detailed in previous literature.^23^

The human wild-type BPB domain deletion version (ΔBPB: amino acids 396–705 deleted) of DNA damage-binding protein 1 (DDB1) (residues 1–1140 Δ396–705, UniProt accession number Q16531) was PCR-amplified from AddGene plasmid #124213 and inserted into a pACEBac1 vector, which had been modified with a N-terminal His_6_ affinity tag. The DDB1ΔB construct was expressed in *Sf9* insect cells and subjected to purification employing a combination of affinity chromatography, anion exchange chromatography, and gel filtration (SEC). Cell pellets were resuspended in lysis buffer (50 mM Tris pH 8, 200 mM NaCl, 1 mM TCEP, 20 mM Imidazole) supplemented with 1 : 100 (v/v) PMSF, 1 : 1000 (v/v) DNase I and cOmplete EDTA-free protease inhibitor cocktail (Roche), followed by lysis through sonication. Cell debris were removed by centrifugation at 70 000 × *g* and 10 °C for 1 h prior to filtration with a syringe filter (0.45 μm). The resulting filtered supernatant was applied onto a HisTrap FF column (Cytiva) connected to an Äkta Start FPLC system, pre-equilibrated with lysis buffer. After loading the supernatant, the column was washed with 10 column volumes (CV) lysis buffer to achieve baseline levels of absorbance at 280 nm wavelength, followed by 3 CV of wash buffer (50 mM Tris pH 8, 200 mM NaCl, 1 mM TCEP, 50 mM Imidazole) and another round of lysis buffer (5 CV). The bound proteins were then eluted using elution buffer (50 mM pH 8, 200 mM NaCl, 1 mM TCEP, 250 mM Imidazole) and peak fractions containing the protein of interest, as assessed by SDS PAGE were combined and concentrated to a volume of 5 mL. The elution sample was diluted 1 : 10 in AIEX start buffer (50 mM Tris pH 8, 1 mM TCEP) to decrease the salt concentration, and then applied to a HiTrap Q HP anion exchange column (Cytiva) connected to a Äkta Start system, pre-equilibrated in AIEX start buffer. Following the loading of the protein sample, bound proteins were eluted using a linear salt gradient, gradually transitioning from AIEX start to AIEX finish buffer (50 mM Tris pH 8, 1 M NaCl, 1 mM TCEP) over 60 mL, ultimately reaching a final ratio of 70% AIEX finish buffer. Protein fractions containing DDB1ΔB protein, as verified by SDS PAGE analysis, were pooled, concentrated and loaded onto a Äkta Pure FPLC system (Cytiva) equipped with a preparative Superdex 200 Increase 10/300 GL column (Cytiva), pre-equilibrated in SEC buffer (50 mM Hepes pH 7.4, 200 mM NaCl, 1 mM TCEP). Peak fractions were monitored by SDS PAGE, and fractions containing homogenous DDB1ΔB protein were pooled, concentrated, aliquoted, snap frozen in liquid nitrogen and stored at −80 °C for downstream applications.

### Protein crystallization and diffraction data collection

DDB1ΔB and Cdk12/CycK proteins in complex with SR-4835 were crystallized using the hanging drop vapor diffusion method at 10 °C. The protein mixture, comprising 80 μM Cdk12/CycK kinase complex, 70 μM of DDB1ΔB, and 5 mM SR-4835, was incubated on ice for 10 min before setting up crystallization drops. Optimal crystals were obtained in 24-well plates (Jena Bioscience) using a 1 : 1 ratio (0.6 μL/0.6 μL) of protein solution and crystallization buffer containing 1 M potassium citrate and 15% glycerol. Hexagonal-shaped crystals appeared within 3–4 days. To enhance crystal stability, 15% ethylene glycol was added to the mother liquor as a cryoprotectant, followed by rapid flash-cooling in liquid nitrogen utilizing Mitegen loops (Mitegen). Data collection up to 3.9 Å resolution was performed at beamline P13 of the Deutsche Elektronen-Synchrotron (DESY, Hamburg, Germany), utilizing an Eiger detector at a wavelength of 0.9763 Å. Data were collected from a single crystal mounted on a loop and maintained at a temperature of 100 K in a stream of cooled nitrogen gas (Oxford Cryosystems).

### Data processing, structure determination, and model building

The collected data underwent processing and scaling using the XDS program package.^34^ The DDB1ΔB·SR-4835·Cdk12/CycK crystals belong to space group *P*3_1_21 with three complexes in the crystallographic asymmetric unit. The phase problem was resolved by the molecular replacement method employing the PHASER program.^35^ The coordinates of DDB1ΔB and Cdk12/CycK (PDB: 6TD3 and 8BU5) served as a search model for the phase solution. Subsequently, the initial model underwent improvements *via* alternating cycles of manual rebuilding and visual assessments in COOT^36^ and refinement using the PHENIX program.^37^ To ensure the stereochemical quality of the model, a Ramachandran plot was employed. The final model encompasses residues 1–1140 Δ395–707 of DDB1ΔB, residues 714–1047 of Cdk12, and residues 20–267 of CycK with one molecule SR-4835 engaged with DDB1ΔB and Cdk12. The model was refined to *R*_work_ and *R*_free_ values of 20.6% and 25.4%, respectively. Detailed information on diffraction data collection, quality, and refinement statistics are given in Table S1 (ESI†). The atomic coordinates and structure factor amplitudes have been deposited in the protein data bank (PDB) under accession code 9FMR.

### Analytical size-exclusion chromatography (HPLC)

Analytical size-exclusion chromatography was employed to investigate compound-induced protein–protein interactions between the heterodimeric Cdk12 (714–1063)/CycK (1–267) complex and the RING E3 ligase-associated adaptor protein DDB1ΔB. The analysis was conducted on an Agilent 1260 Infinity II HPLC system (Agilent Technologies), coupled with a Superdex 200 Increase 3.2/300 column (Cytiva), pre-equilibrated in Cdk12 SEC buffer (20 mM Hepes pH 7.8, 400 mM NaCl, 1 mM TCEP) supplemented with 1 g L^−1^ carboxymethyl-dextran. Purified proteins, combined in equimolar ratios (20 μM Cdk12/CycK and DDB1ΔB, respectively) were supplemented with 3-fold molar excess of small molecular compounds. The resulting protein mixtures were diluted to a final volume of 25 μL using the kinases SEC-buffer ensuring a final DMSO concentration of 2%, followed by an incubation period of 10 min. Upon injection into the analytical system, the chromatography was conducted at a controlled flow rate of 0.05 mL min^−1^, with protein absorbance monitored at 280 nm wavelength. Peak fractions were collected drop by drop and subsequently subjected to analysis *via* dynamic light scattering (DLS) to assess their size distribution, along with SDS-PAGE analysis to determine the composition of the protein complex.

### Dynamic light scattering (DLS)

To evaluate particle size distribution in liquid solution, dynamic light scattering was employed. Specifically, 10 μL fractions from the central region of peaks obtained in analytical size exclusion chromatography runs were analyzed. This analysis was performed using a DynaPro NanoStar 672 (Wyatt Technology) in a disposable DLS MicroCuvette (Wyatt Technology), while maintaining a constant temperature of 25 °C. Each measurement was conducted with an acquisition time of 3 s, and each data point depicted in the Results section represents the average of 20 individual DLS measurements. The hydrodynamic radius of the protein samples was determined using the processing and analysis software DYNAMICS.

### Thermal protein stability analysis (nanoDSF)

To investigate the thermal stability of proteins and protein complexes in solution, as well as to examine the impact of small molecular compounds on the proteins stability, the nano-differential scanning fluorimetry (nanoDSF) method was employed using a Prometheus NT.48 (NanoTemper) device. In thermal shift assays, proteins were diluted to a final concentration of 5 μM in SEC buffer (50 mM Hepes pH 7.4, 200 mM NaCl, 1 mM TCEP), supplemented with either 2% DMSO or a 3-fold molar excess of the small molecular compound, ensuring a final DMSO concentration of 2%. For monitoring ternary complexes, DDB1ΔB and Cdk12/CycK proteins were mixed in equimolar ratios (5 μM) in SEC buffer in the presence of 3-fold molar excess of the compound. A 10 min incubation preceded measurements in Prometheus NT.48 Standard Capillaries (NanoTemper). Thermal stability was monitored over a temperature range of 20–90 °C with a linear gradient of 1.5 °C min^−1^, utilizing the PR.ThermControl software (NanoTemper). The melting temperature (*T*_m_), represented by maxima/minima of the first derivative of the 350/330 nm ratio, was extracted by the PR.ThermControl software. All measurements were conducted at least in quadruplets.

### Pulldown assay (University of Bonn)

The pulldown assay was utilized to identify drug-triggered protein–protein interactions involving the Cdk12 (714–1063)/CycK (1–267) complex and the DDB1ΔB protein. In this assay, purified GST-tagged Cdk12 construct complexed with GST-CycK was combined with equimolar amounts of His_6_-tagged DDB1ΔB (5 μM) in the presence of 5 μM of the respective small molecular compound in pulldown assay buffer (50 mM Hepes pH 7.4, 200 mM NaCl, 0.25 mM TCEP, 0.05% (v/v) Tween20), ensuring a 2% total DMSO concentration. Experimental controls included samples supplemented with 2% DMSO instead of compound as well as samples devoid of the GST-tagged Cdk12/CycK complex. Each sample was individually added to approximately 50 μL of pre-equilibrated glutathione agarose beads (Thermo Fisher Scientific). The bead suspensions were then subjected to a 2 h incubation period at room temperature with gentle agitation. After incubation, the beads were pelleted by centrifugation at 700 × *g* for 4 min, the supernatant was discarded, and the beads were washed three-times with 100 μL of pulldown assay buffer. Following the washing step, bound proteins were eluted from the beads with 50 μL of pulldown assay buffer supplemented with 10 mM GSH for 1 h at room temperature under rotation. Subsequently, the beads were again pelleted by centrifugation at 700 × *g* for 4 min and the resulting supernatants containing eluted protein fractions were mixed with 6 × SDS sample buffer at a 1 : 1 (v/v) ratio. The eluted protein samples were separated by SDS-PAGE and subjected to Coomassie brilliant blue staining for visualization and analysis.

### Microscale thermophoresis (MST)

A protocol to assess ternary binding of molecular glues with CDK12/cycK and DDB1 by MST was established using published procedures for PROTAC interaction with bromodomains and E3 ubiquitin ligases.^38^ His_8_-DDB1-ΔBPB was labeled with the MO-L018 Monolith™ His-tag Labeling Kit RED-tris-NTA 2nd Generation (NanoTemper Technologies) following the instruction manual. 100 nM dye was mixed with 200 nM DDB1 in 50 mM HEPES pH 7.5, 150 mM NaCl, 5% glycerol, 0.05% Tween, and incubated at room temperature for 30 min. Experiments were performed using a constant concentration of labeled DDB1 (10 nM), and increasing concentrations of compounds using a 16 point 2× serial dilution (0.076 nM to 2500 nM or 0.763 nM to 25 000 nM depending on the *K*_d_ range) with or without CDK12/cyclin K (1000 nM) in 50 mM HEPES pH 7.5, 150 mM NaCl, 5% glycerol, 0.05% tween and 5% DMSO, 0.5 mM DTT. After 5 min incubation at room temperature, the samples were loaded in MO-K022 standard treated capillaries (NanoTemper Technologies) and measured by a Monolith NT.115 Pico instrument (NanoTemper Technologies) at 40% MST power and 20% excitation power (auto-detect) on Pico-Red detector at 22 °C. MST traces were recorded using standard parameters: 5 s MST power off, 30 s MST power on and 5 s MST power off. Measurements were taken at −1 to 0 s (cold region) and 9–10 s (hot region). Data were analyzed using a fitting function derived from the law of mass action:1
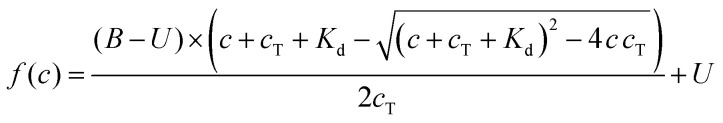
where *f*(*c*) is the fluorescence signal (*F*_norm_) at a given ligand concentration (*c*), *B* is *F*_norm_ of fully liganded DDB1 at high ligand concentrations, *U* is *F*_norm_ of DDB1 alone at low ligand concentrations, *K*_d_ is the dissociation constant or binding affinity, *c*_T_ is the final concentration of DDB1 in the assay (Fig. 3G). For normalization, the data were first transformed using eqn (2) where y is the fraction bound, followed by fitting to eqn (3) (Fig. 5B–E).2
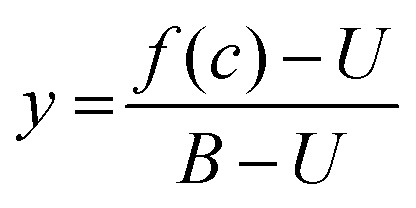
3
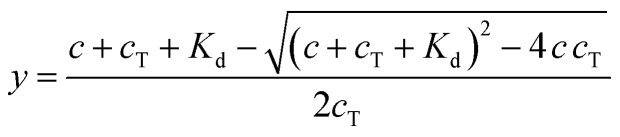


### Pulldown protocol

Biotinylation was achieved by conjugating azide-PEG3-biotin to the photoactivable probe (SR-4784-PAP) using the click reaction procedure as described earlier.^27^ Briefly, 20 μL of 5 mM azide-PEG3-biotin (Sigma-Aldrich, 762024-25MG) was added to 1 mg mL^−1^ cell lysate aliquots. To every aliquot a mix of 60 μL 1.7 mM, TBTA, 20 μL 50 mM CuSO_4_ and 20 μL 50 mM TCEP and incubated for 2 h at room temperature. Then, the lysates were purified by size exclusion chromatography by SpinOUT G-600 columns (G-Biosciences, 786–1621). The purified lysates were incubated with neutravidin coupled agarose beads (Thermo Fisher Scientific) for overnight at 4 °C. The beads were washed several times with Tween-TBS buffer, resuspended in elution buffer (HEPES pH 8; 50 mM, NaCl 150 mM, EDTA pH 8; 5 mM, SDS 4%), and incubated for 30 minutes at 80 °C. Beads were pelleted, and the eluates were resolved by western blotting.

### Plasmid construction, lentiviral packaging and generation of stable gene knockdown cell lines

For stable genetic knock-down of DDB1 in cells, predesigned and validated TRC2-pLKO-puro shRNA plasmids for DDB1 (TRCN0000082855 and TRCN0000082856), and a non-targeting shRNA plasmid (SHC001) were obtained from Millipore Sigma. All plasmids were purified using the Qiagen Maxiprep kit (Qiagen). FuGENE HD 6 transfection reagent (Promega) was used for transfection of plasmids and lentiviral gene/shRNA constructs. Mission lentiviral packaging mix (Millipore Sigma) was used to package lentiviral plasmids into lentiviral particles in HEK293T cells, as per manufacturer's protocols. The resultant virus was concentrated using Vivaspin 20 centrifugal concentrators (Sartorius). MDA-MB-231 and HEK293T cells were transduced with lentivirus. Stable knocked down DDB1 (shDDB1-231 and shDDB1-HEK293T) cells were generated after selection with the antibiotic puromycin, and genetic knockdown was confirmed by western blotting.

### Transient gene knockdown studies

Gene-specific Silencer® Select siRNAs for human DDB1, Cullin4A, Cullin4B, VHL, Cereblon (CBRN) as well as Silencer® Select negative control siRNA (Thermo Fisher Scientific) were resuspended in nuclease-free water and stored at −20 °C. Cells were transfected with individual siRNA or a combination of siRNAs (for dual knockdown) diluted in serum free Opti-MEM medium (Gibco) with Lipofectamine RNAi MAX Reagent (Thermo Fisher Scientific) as per manufacturer's recommendations. Transfected cells were treated with SR-4835 (0.5 μM) for time points indicated in the figure legends. After 48 hours of transfection, cells were lysed, and the silencing efficiency of the siRNAs were confirmed by SDS-PAGE and western blotting.

### CRISPR/cas9 editing

CRISPR knockout plasmids were created by cloning gene-specific guide RNA sequences into the lentiCRISPR v2 vector (Addgene #52961). HEK293T cells were transduced with the CRISPR knockout plasmids and packaging plasmids (MISSION system, Sigma Aldrich) using FuGENE HD (Promega) according to the manufacturer's instructions. Twenty-four hours after transfection, the medium was changed, and virus-containing media were collected at 72 hours post-transfection and filtered. Target cells were then transduced with the lentivirus-containing media and selected with puromycin. Following selection, CRISPR knockout cells were harvested to check knockout efficiency and expanded for mechanistic studies.

### SDS-PAGE and western blotting

Total cell lysates were prepared using RIPA lysis buffer (Millipore Sigma) supplemented with protease and phosphatase inhibitors (Roche). Protein concentrations were estimated using the BCA protein assay kit (Pierce, Thermo Fisher Scientific). Equal amounts of proteins were separated using NuPAGE 4–12% Bis-Tris gels (Thermo Fisher Scientific). For CLICK chemistry pulldown experiments, proteins in the eluates were resolved using 3–8% Tris–acetate gels (Thermo Fisher Scientific). Proteins were transferred to nitrocellulose membranes using NuPAGE transfer buffer (Thermo Fisher Scientific). Membranes were blocked in LI-COR blocking buffer for 1 hour at room temperature and then probed with primary antibodies diluted in the same blocking buffer overnight at 4 °C. To detect the primary antibodies, membranes were incubated with IR-680 or IR-800 conjugated secondary antibodies (LI-COR) diluted in LI-COR blocking buffer at room temperature for 1 hour. Washes with 0.01% Tween-phosphate buffer were performed after primary and secondary antibody incubation steps. Signals from the membrane were recorded by scanning on a LI-COR Odyssey imager. Bands were quantified using Image Studio Lite (LI-COR Biosciences) or the ImageJ program.

### DC_50_ analysis

#### By immunoblotting

Cells were treated with SR-4835 (3 μM, 1 μM, 0.3 μM, 0.1 μM, 0.03 μM and 0.01 μM) for 1 hour. To visualize the degradation of cyclin K, total cell lysates were subjected to SDS-PAGE and western blotting.

#### By HiBiT assay

The HiBiT-CCNK KI A549 cells (Promega) were seeded in 384 well plate and treated with the test compounds for 2 hours in a dose dependent manner. Nano-Glo® HiBiT Lytic Detection System (Promega) composed of the Nano-Glo® HiBiT Lytic Substrate, Nano-Glo® HiBiT Lytic Buffer and LgBiT Protein was used as per the manufacturer's instructions. Briefly, a 2× Nano-Glo® HiBiT Lytic Detection Reagent mixture was prepared by adding 20 μL of Nano-Glo® HiBiT Lytic Substrate and 10 μL of LgBiT protein per every 1 mL of Nano-Glo® HiBiT Lytic Buffer. After incubating the cells with the 2× Nano-Glo® HiBiT Lytic Detection Reagent mixture, luminescence was measured. HiBiT tagged cyclin K present at any given concentration was measured by normalizing the luminescence signal at that concentration to the luminescence signal from the DMSO (control) cells. The concentration necessary to achieve a 50% reduction in cyclin K levels (DC_50_) and the maximum achievable reduction in cyclin K levels (*D*_max_) was computed from this assay.

### Cell viability assay

To assess inhibition of cellular viability after drug treatment, 500 cells per well were seeded in a 384-well plate (Corning, USA) and treated for 72 hours with increasing concentrations of the test compounds. Cell viability was measured by Cell Titer Glo assay kit (Promega, USA). EC_50_ was determined from the nonlinear regression and a four-parameter algorithm on the GraphPad Prism software.

### Immunofluorescence

Cells were fixed with 4% PFA in PBS at room temperature, permeabilized with 0.1% Triton X-100 in PBS, and blocked with 3% BSA in PBS at room temperature. Cells were incubated with primary antibodies overnight at 4 °C, followed by incubation with Alexa-Fluor conjugated secondary antibodies for 1 hour at room temperature. Cell nuclei were stained using DAPI. Images were acquired on a confocal microscope (Leica). ImageJ and QuPath were used for image analysis.

### Compound pull-down and shotgun proteomics

The breast cancer cells (MDA-MB-231) were treated with sub-IC50 doses (as indicated in the figure) of four molecular glue-like compounds generated from the same CDK12 scaffold for 2 hours. The cells were then washed three times with cold PBS. Cells were lysed in aqueous denaturing buffer containing 8 M urea, 20 mM HEPES (pH 8), 1 mM sodium orthovanadate, 2.5 mM sodium pyrophosphate and 1 mM β-glycerophosphate. Bradford assays determined the protein concentration for each sample. Protein disulfides were reduced with 4.5 mM dithiothreitol (DTT) at 60 °C for 30 minutes and then cysteines were alkylated with 10 mM iodoacetamide for 20 minutes in the dark at room temperature. Trypsin digestion was carried out at room temperature overnight with an enzyme-to-substrate ratio of 1 : 20, and tryptic peptides were acidified with aqueous 1% trifluoroacetic acid (TFA) and desalted with C18 Sep-Pak cartridges according to the manufacturer's procedure. An aliquot (100 mg) of each tryptic digest was tandem mass tag (TMTpro 18plex) labeled. The TMT channel layout is: DMSO samples: 126C, 127C and 128C. MR1014 samples: 127N, 128N and 129N. MR1110 samples: 129C, 130C and 131C. MR1187 samples: 130N, 131N and 132N. MR1226 samples in 132C, 133C and 134C. SR4835 samples: 133N, 134N and 135N. Label incorporation in each channel was verified to be >95% by LC-MS/MS and spectral counting. The samples were then pooled and lyophilized.

After lyophilization, TMT-labeled peptides were redissolved in 200 mL of aqueous 20 mM ammonium formate, pH 10.0. The basic pH reversed-phase liquid chromatography (bRPLC) separation was performed on an XBridge 4.6 mm ID × 100 mm length column packed with BEH C18 resin, 3.5 μm particle size, 130 Å pore size (Waters). The bRPLC Solvent A was aqueous 2% ACN with 5 mM ammonium formate, pH 10.0. Peptides were eluted by using the following gradient program: 5% bRPLC B (aqueous 90% acetonitrile with 5 mM ammonium formate, pH 10.0) for 10 minutes, 5–15% B in 5 minutes, 15–40% B in 47 minutes, 40–100% B in 5 minutes and 100% B held for 10 minutes, followed by re-equilibration at 1% B. The flow rate was 0.6 mL min^−1^, and 24 concatenated fractions were collected for expression proteomics. Vacuum centrifugation (Speedvac, Thermo) was used to dry the samples.

Liquid chromatography-tandem mass spectrometry was performed using a Thermo Easy nLC 1200 and Thermo Q Exactive HF-X mass spectrometer for peptide sequencing and TMT readout. Peptides were loaded on a Acclaim C18-PepMap100 trapping column (75 μm ID × 2 cm length, 3 μm particle size, 100 Å pore size) and separated using a 300 nL min^−1^ flow rate on an Acclaim Easy Spray C18-PepMap100 Column (75 μm ID × 25 cm length, 2 μm particle size, 100 Å pore size) using a 120 minute gradient with an initial starting condition of 2% B buffer (0.1% formic acid in 90% acetonitrile) and 98% A buffer (0.1% formic acid in water). Buffer B was increased to 28% over 90 minutes, then up to 40% in an additional 10 minutes. High B (90%) was run for 15 minutes to wash the column. The mass spectrometer was outfitted with a Thermo EASYSpray source with the following parameters: Spray voltage: 2.0, Capillary temperature: 275 °C, Funnel RF level: 40. Parameters for data acquisition were: MS resolution was 60 000 with an AGC target 3e6 and max IT time 50 ms, the *m*/*z* range was set as 400–1600 Th. MS/MS data were acquired with resolution 15 000, AGC 1e5, max IT 50 ms, and the top 30 peaks were picked with an isolation window of 1.6 *m*/*z* around the monoisotopic peak with a dynamic execution of 25 s.

### Data analysis of the shotgun proteomics

MaxQuant (version 1.6.14.0) was used to identify peptides using the UniProt human database (March 2022) and quantify the TMT reporter ion intensities. Up to 2 missed trypsin cleavages were allowed. The mass tolerance was 20 ppm for the first search and 4.5 ppm for the main search. Reporter ion mass tolerance was set to 0.003 Da. Minimal Precursor intensity fraction was set to 0.75. Carbamidomethyl cysteine was set as a fixed modification. Phosphorylation on serine/threonine/tyrosine and methionine oxidation were set as variable modifications. Both peptide spectral match (PSM) and protein false discovery rate (FDR) were set at 0.01. The match between runs feature was activated to carry identifications across samples. Similar parameters were used for Mascot searches to support the data upload. Data were log_2_ transformed for analysis in Microsoft Excel. Normalization was performed using geometric mean. To compare each degrader to control, the log_2_ ratio values and *p* values from student's *t*-tests were calculated to assess the magnitude and consistency of differences, respectively. Differences were considered significant if the absolute value of the log_2_ ratio value exceeded 2 standard deviations from the average log_2_ ratio for that comparison.

### Data collection and statistical analysis

Statistical analyses and graphical presentations were performed using Prism 7.0 (GraphPad). Statistical assays performed are specified in figure legends.

## Data availability

Proteomics data and bioinformatic analysis pipeline is available *via* ProteomeXchange with identifier PXD053505. The crystallographic data for SR-4835 has been deposited in the Protein Data Bank (PDB) under the accession number 9FMR.

## Conflicts of interest

The authors declare the following financial interests/personal relationships which may be considered as potential competing interests: A. M. D. D. and W. R. have a patent issued to The Scripps Research Institute and University of Florida (US11666578B2). The authors declare no other financial interests or personal relationships that could be considered potential competing interests.

## Supplementary Material

CB-OLF-D4CB00190G-s001
