# Supplementary material for: Discovery and design of molecular glue enhancers of CDK12–DDB1 interactions for targeted degradation of cyclin K
Source: RSC Chem Biol. 2024 Oct 11;6(1):36–55. doi: 10.1039/d4cb00190g (PMC11494886; doi:10.1039/d4cb00190g)
Supplement: CB-006-D4CB00190G-s001 [file CB-006-D4CB00190G-s001.pdf]

Supplementary Information (SI) for RSC Chemical Biology.
This journal is © The Royal Society of Chemistry 2024

Supplementary figures

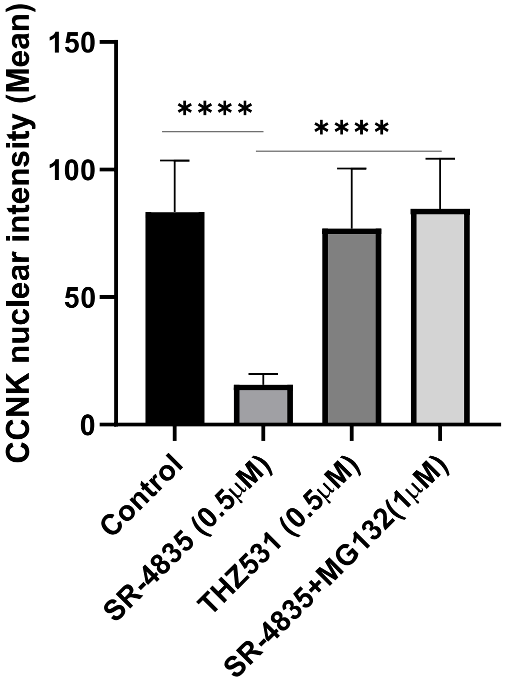

Figure S1. Quantification of changes in the cyclin K nuclear intensity. Error bars indicate standard deviation (SD). Comparison between the indicated groups was performed by two-tailed Student's t-test which shows significant difference in the levels of cyclin K by SR-4835 treatment and the rescue of SR-4835 induced cyclin K degradation by MG132 (****, $p < 0.0001$).

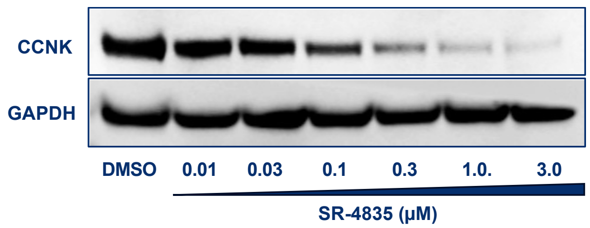

Figure S2. Immunoblot depicting cyclin K levels in MDA-MB-231 cells treated with the indicated concentration of SR- 4835.

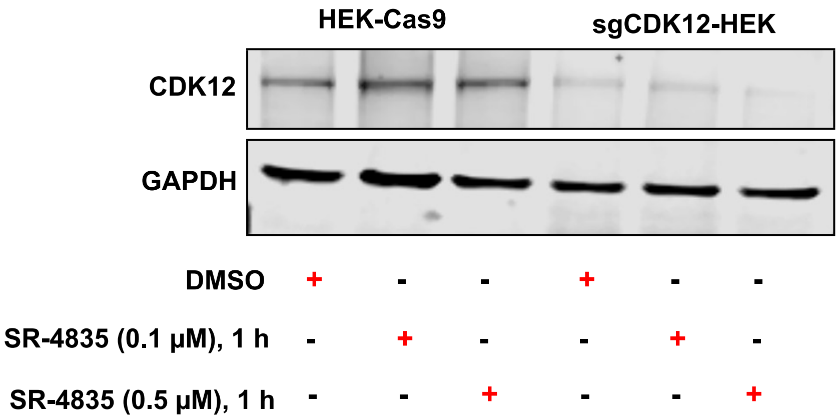

Figure S3. Immunoblot depicting CDK12 knockout by CRISPR/Cas9 in HEK-293T cells. SR-4835 treated cells (as indicated) show no change in CDK12 levels.

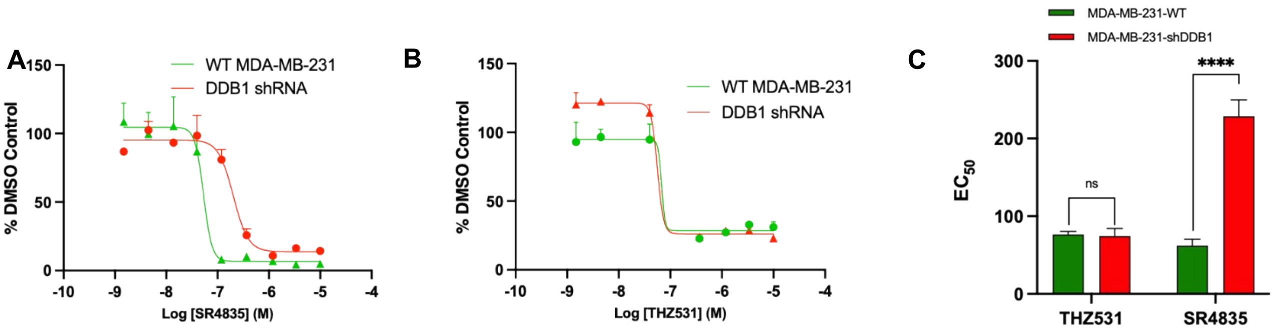

SR-4835	EC_{50}	fold change
WT MDA-MB-231	55	
DDB1 shRNA	218	3.9

THZ-531	EC_{50}	fold change
WT MDA-MB-231	72	
DDB1 shRNA	65	0.9

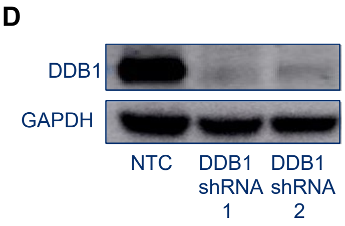

Figure S4. The molecular glue activity of SR-4835 is correlated with its cytotoxicity in MDA-MB-231

(A, B) CellTiter-Glo® viability assays comparing the EC_{50} values of SR-4835 and THZ531 in MDA-MB-231 cells and its isogenic MDA-MB-231 paired cell line with downregulated DDB1 expression (MDA-MB-231$_{DDB1\text{-}shRNA}$).

(C) Quantification of difference found in the EC_{50s} of THZ531 and SR-4835 in the MDA-MB-231 and MDA-MB-231$_{DDB1\text{-}shRNA}$ cells after 72 hours. Error bars indicate standard deviation (SD). Comparison between the indicated groups was performed by two-tailed Student's t-test (****, $p < 0.0001$).

(D) Immunoblot showing stable knockdown of DDB1 in MDA-MB-231 cell lines

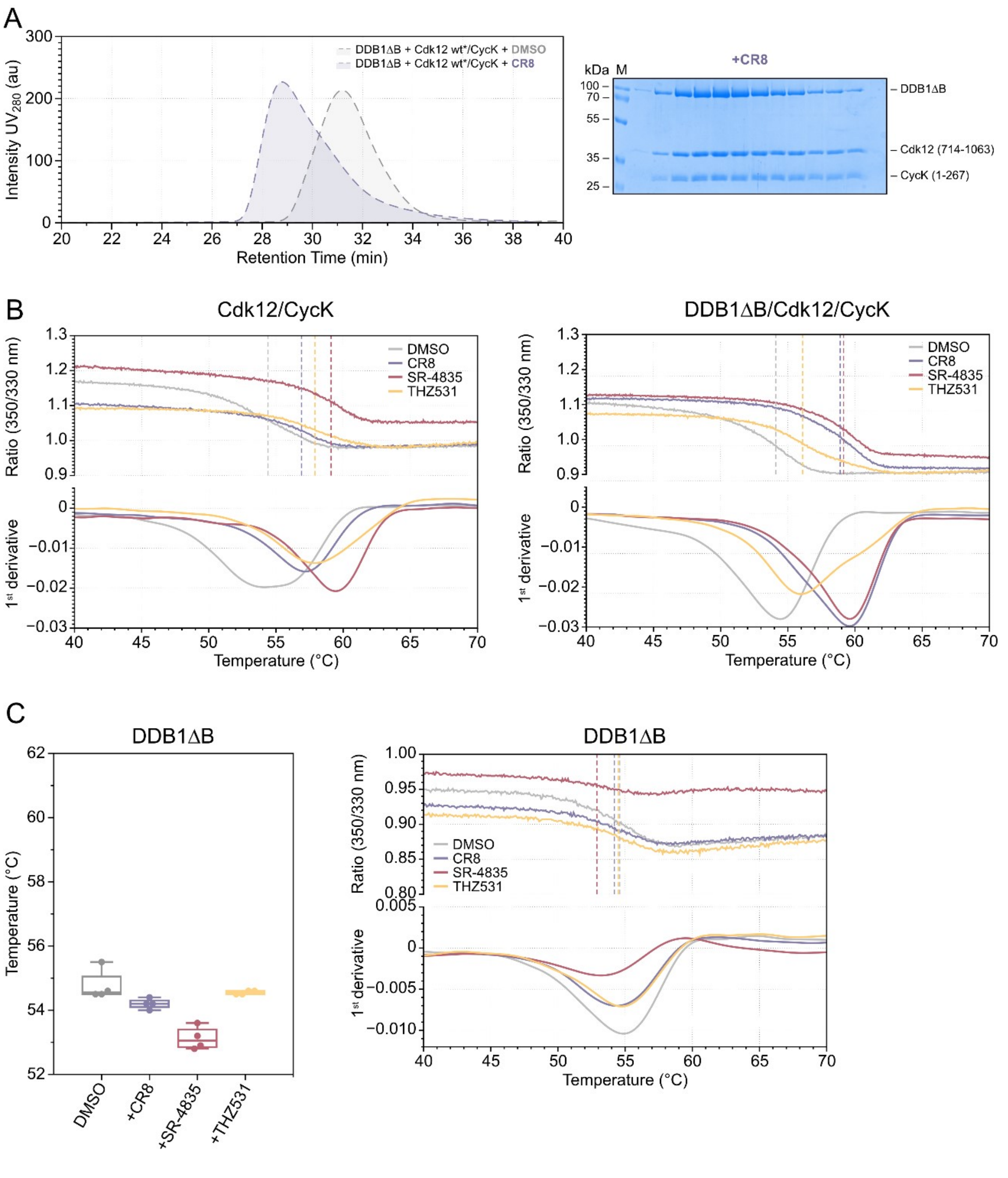

Figure S5. (A) Analytical size exclusion chromatography of purified, recombinant DDB1ΔB and Cdk12/CycK proteins (20 μM) in the presence of either 2% DMSO or 60 μM small molecular compounds CR8. (B)

A

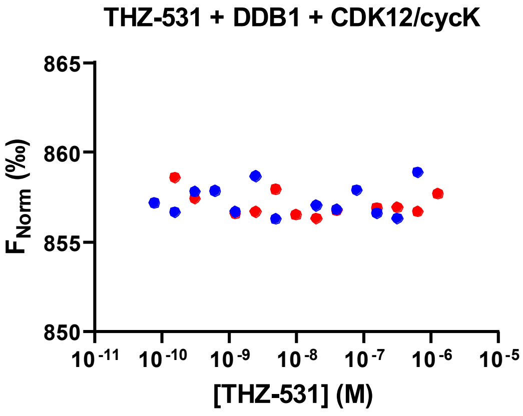

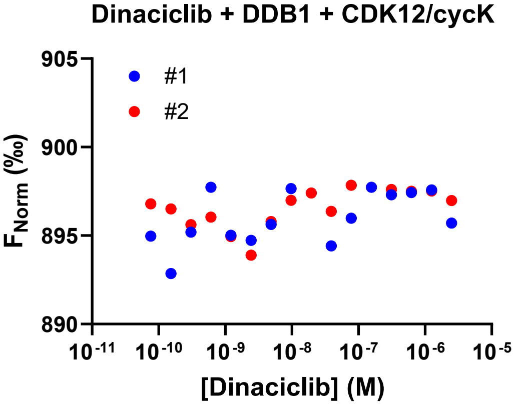

B

D

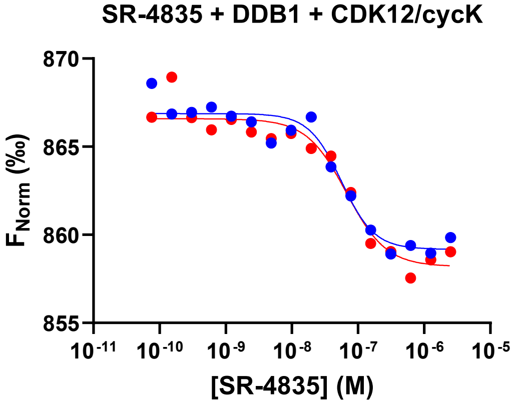

C

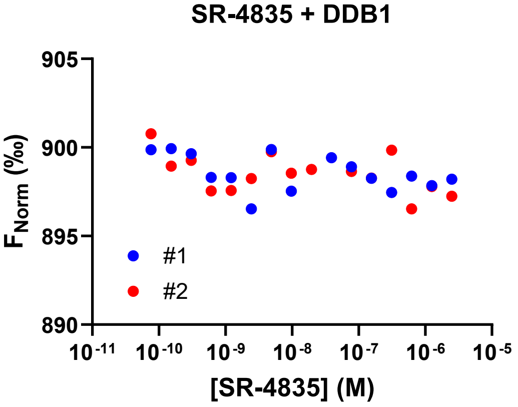

Figure S6. Quantification of tertiary complex formation of CDK12/cyclin K + DDB1+SR-4835/THZ531 using microscale thermophoresis. Microscale thermophoresis studies showing that THZ-531 and dinaciclib lack the ability to induce a ternary complex with CDK12/cycK and DDB1 (A,B), and that SR-4835 lacks the ability to interact with DDB1 in the absence of CDK12/cycK (C, D) (duplicate measurements).

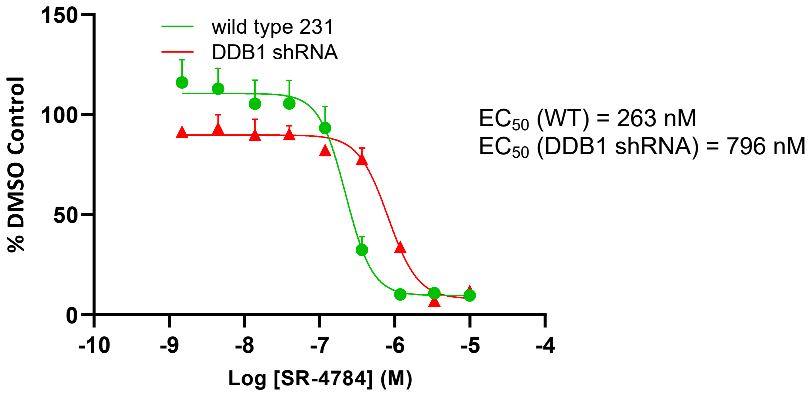

Figure S7. Cell viability of SR-4784 in MDA-MB-231 wild type and MDA-MB-$231_{DDB1\text{-}shRNA}$

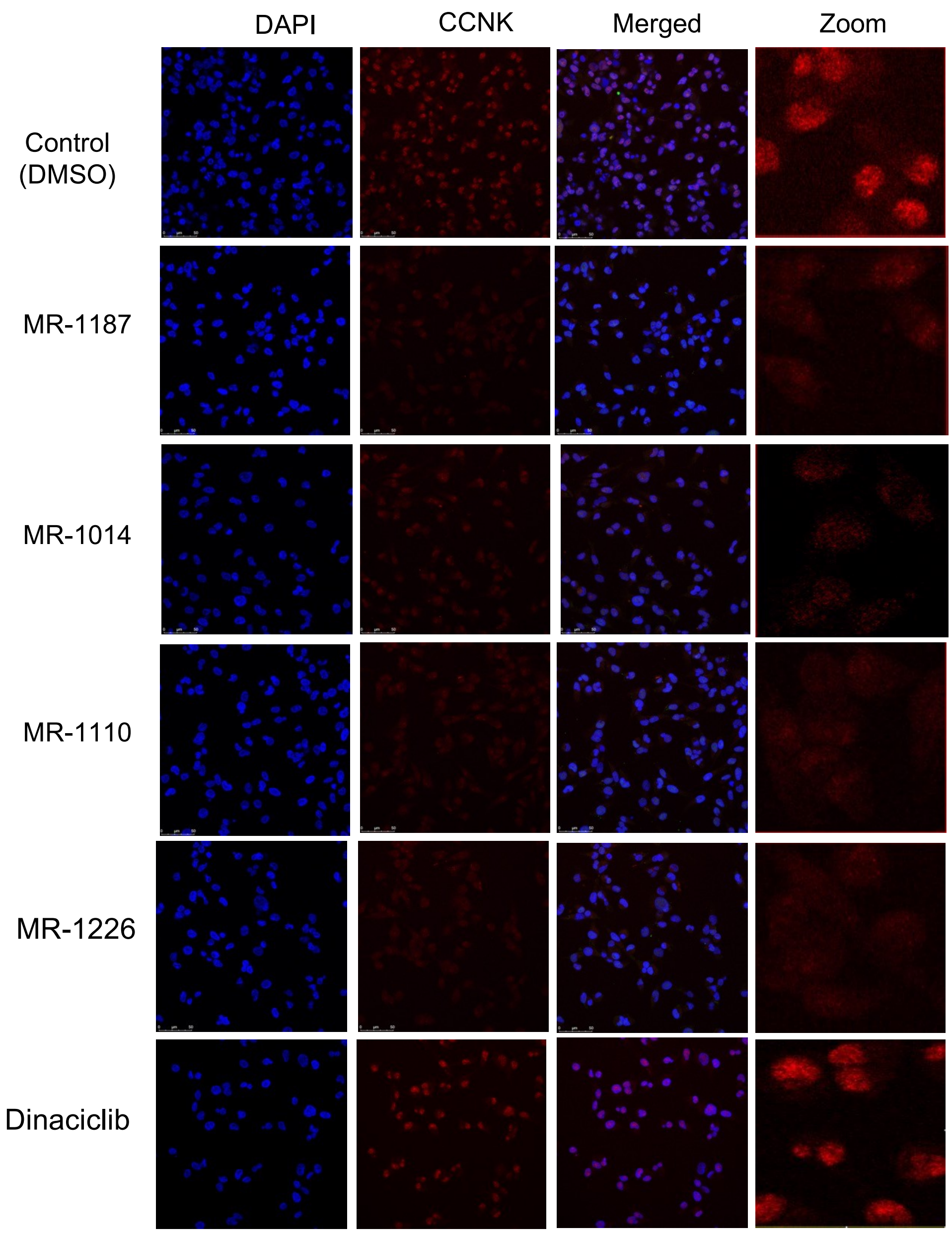

Figure S8. Immunofluorescence staining for cyclin K degradation. MDA-MB-231 cells were treated for 2 h with either DMSO or the indicated compounds (0.5 µM) . Cells were fixed and stained with Cyclin K antibody followed with Alexafluor 555 (red) conjugated secondary antibody. Nuclei were stained with DAPI (blue).

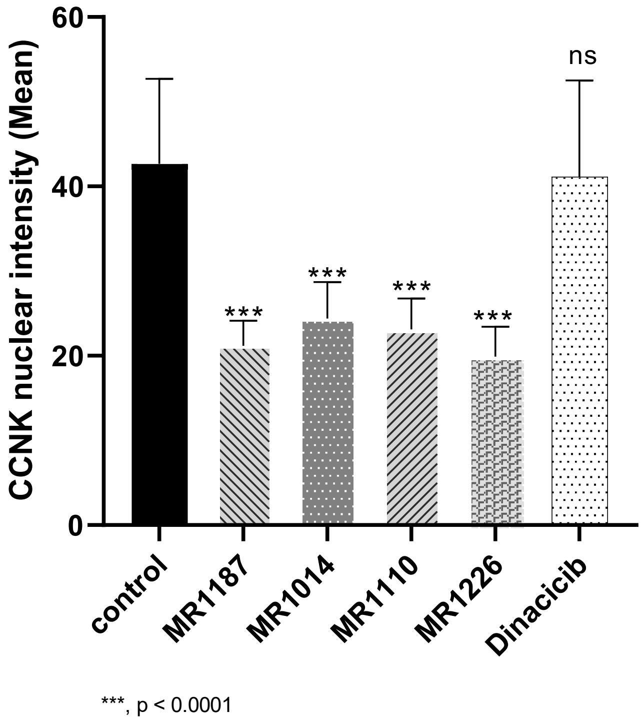

Figure S9. Quantification of changes in the cyclin K nuclear intensity. Error bars indicate standard deviation (SD). Comparisons between the control and the individual groups were performed by the two-tailed Student's t-test showing that compared to the control, the cyclin K levels are reduced by MR-1187, MR-1014, MR-1110 and MR-1226 (***, $p < 0.0001$), but not affected by dinaciclib.

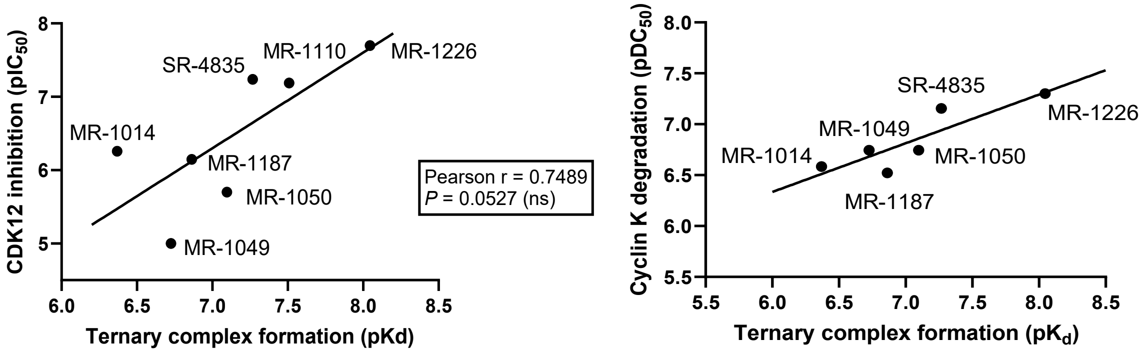

Figure S10. Pearson correlation analysis between ternary complex formation and inhibition of CDK12 (left panel) or degradation of cyclinK (right panel). *P* values < 0.05 were considered significant.

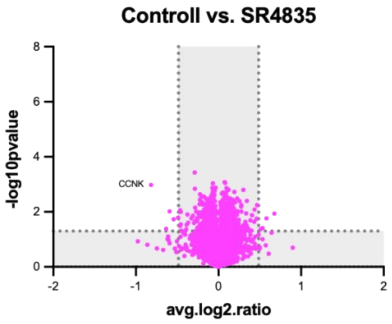

Figure S11. Label-free TMT-based quantitative proteomics upon treatment with SR-4835 at 6 h. Plot shows the significantly differentially abundant proteins ranked according to their p value (Y-axis) as -log 10 and their relative abundance Log2 ratio (X axis) between DMSO control and the compound of interest. All samples were generated in triplicates and 0.5 fold changes cut off were used for avg log2 ratio's to analyze the data. The p values < 0.05 were considered as significant.

MR-1015

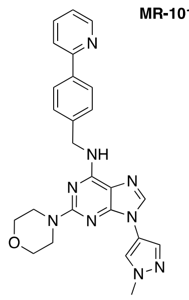

CDK12 IC_{50} = 2.6 μM
CCNK DC_{50} = > 5 μM
MDA-MB231 GI_{50} = 5.8 μM

Figure S12. Structure of phenylpyridine analog MR-1015 and its activity in CDK12 functional and cell-based assays.

Supplementary Table 1: Data collection and refinement statistics

	**DDB1ΔB•SR-4835•Cdk12/CycK**
Data collection[a]	
Beam line	P13, DESY
Wavelength [Å]	0.9763
Space group	P 3_1 2 1
Unit cell: a, b, c [Å]	249.521, 249.521, 218,535
α, β, γ [°]	90, 90, 120
Resolution range [Å]	36.57-3.9 (4.04-3.9)
Unique reflections	71619 (1811)
Multiplicity	19.6 (20.5)
Completeness (%)	91.42 (25.63)
Mean I/sigma(I)	5.90 (0.20)
R_{meas}	0.4128 (7.311)
$CC_{1/2}$	0.997 (0.117)
Reflections used in refinement	65570 (1811)
Reflections used for R-free	2008 (55)
Refinement	
Model content	A: DDB1ΔB (1-1140 Δ395-707)<br>B: Cdk12 (714-1047), SR-4835<br>C: CycK (20-267)
# of atoms macromolecules	33685
# of ligands	102
# of solvent	3
R_{work}	0.2064 (0.4069)
R_{free}	0.2546 (0.3958)
RMS deviations bonds [Å]	0.009
RMS deviations angles [°]	0.83
Ramachandran favored (%)	95.2
Ramachandran allowed (%)	4.52
Ramachandran outliers (%)	0.29
Rotamer outliers (%)	2.00
Clashscore	6.35
Average B-factor	243.22
Macromolecules	243.27
Ligands	234.28
Solvent	30.00
PDB accession code	9FMR
